# Recurrent XPO1 mutations alter pathogenesis of chronic lymphocytic leukemia

**DOI:** 10.1186/s13045-021-01032-2

**Published:** 2021-01-15

**Authors:** Janek S. Walker, Zachary A. Hing, Bonnie Harrington, Jordan Baumhardt, Hatice Gulcin Ozer, Amy Lehman, Brian Giacopelli, Larry Beaver, Katie Williams, Jordan N. Skinner, Casey B. Cempre, Qingxiang Sun, Sharon Shacham, Benjamin R. Stromberg, Matthew K. Summers, Lynne V. Abruzzo, Laura Rassenti, Thomas J. Kipps, Sameer Parikh, Neil E. Kay, Kerry A. Rogers, Jennifer A. Woyach, Vincenzo Coppola, Yuh Min Chook, Christopher Oakes, John C. Byrd, Rosa Lapalombella

**Affiliations:** 1 Division of Hematology, Department of Internal Medicine, The Ohio State University, 460 OSUCCC, 410 West 12th Avenue, Columbus, OH 43210 USA; 2 Department of Veterinary Biosciences, The Ohio State University, Columbus, OH USA; 3 Department of Pharmacology, University of Texas Southwestern Medical Center, Dallas, TX USA; 4 Department of Biomedical Informatics, The Ohio State University, Columbus, OH USA; 5 Center for Biostatistics, Department of Biomedical Informatics, The Ohio State University, Columbus, OH USA; 6 Department of Pathology and State Key Laboratory of Biotherapy, West China Hospital, Sichuan University, Chengdu, 610041 China; 7 Karyopharm Therapeutics Inc, Newton, MA USA; 8 Department of Radiation Oncology, Arthur G James Comprehensive Cancer Center and Richard L. Solove Research Institute, The Ohio State University, Columbus, OH USA; 9 Department of Pathology, The Ohio State University, Columbus, OH USA; 10 Department of Medicine, Division of Hematology, University of California-San Diego School of Medicine, San Diego, CA USA; 11 Division of Hematology, Department of Medicine, Mayo Clinic, Rochester, MN USA; 12 Department of Cancer Biology and Genetics, The Ohio State University College of Medicine, Columbus, OH USA; 13 Genetically Engineered Mouse Modeling Core, The Ohio State University and Arthur G. James Comprehensive Cancer Center, Columbus, OH USA; 14 Division of Medicinal Chemistry, College of Pharmacy, The Ohio State University, Columbus, OH USA

**Keywords:** XPO1, Chronic lymphocytic leukemia, Mouse model, Selinexor, Sines, Expression profiling, Mutation analysis

## Abstract

**Background:**

Exportin 1 (XPO1/CRM1) is a key mediator of nuclear export with relevance to multiple cancers, including chronic lymphocytic leukemia (CLL). Whole exome sequencing has identified hot-spot somatic *XPO1* point mutations which we found to disrupt highly conserved biophysical interactions in the NES-binding groove, conferring novel cargo-binding abilities and forcing cellular mis-localization of critical regulators. However, the pathogenic role played by change-in-function *XPO1* mutations in CLL is not fully understood.

**Methods:**

We performed a large, multi-center retrospective analysis of CLL cases (*N* = 1286) to correlate nonsynonymous mutations in *XPO1* (predominantly E571K or E571G; *n* = 72) with genetic and epigenetic features contributing to the overall outcomes in these patients. We then established a mouse model with over-expression of wildtype (wt) or mutant (E571K or E571G) *XPO1* restricted to the B cell compartment (Eµ-XPO1). Eµ-XPO1 mice were then crossed with the Eµ-TCL1 CLL mouse model. Lastly, we determined crystal structures of XPO1 (wt or E571K) bound to several selective inhibitors of nuclear export (SINE) molecules (KPT-185, KPT-330/Selinexor, and KPT-8602/Eltanexor).

**Results:**

We report that nonsynonymous mutations in XPO1 associate with high risk genetic and epigenetic features and accelerated CLL progression. Using the newly-generated Eµ-XPO1 mouse model, we found that constitutive B-cell over-expression of wt or mutant *XPO1* could affect development of a CLL-like disease in aged mice. Furthermore, concurrent B-cell expression of *XPO1* with E571K or E571G mutations and *TCL1* accelerated the rate of leukemogenesis relative to that of Eµ-TCL1 mice. Lastly, crystal structures of E571 or E571K-XPO1 bound to SINEs, including Selinexor, are highly similar, suggesting that the activity of this class of compounds will not be affected by *XPO1* mutations at E571 in patients with CLL.

**Conclusions:**

These findings indicate that mutations in *XPO1* at E571 can drive leukemogenesis by priming the pre-neoplastic lymphocytes for acquisition of additional genetic and epigenetic abnormalities that collectively result in neoplastic transformation.

## Introduction

Chronic lymphocytic leukemia (CLL) is the most prevalent adult leukemia in Western societies, characterized by clonal expansion of mature CD5^+^/CD19^+^ expressing B lymphocytes with significant genetic and clinical heterogeneity [1]. The majority of CLL patients are diagnosed at an early stage with low tumor volume and moderate disease activity [2]. While a small percentage of CLL cases will never progress from this indolent disease phase, most patients will maintain a watchful waiting period until worsening cytopenias or disease symptoms emerge, upon which treatment begins. Recent advances in treatment of patients with CLL have dramatically improved patient outcomes, prompted by innovations in drugs targeting B-cell receptor (BCR) signaling pathways including Bruton’s tyrosine kinase (BTK) [3–8] or phosphoinositide 3-kinases (PI3K) [9–13], or disruption of the B-cell lymphoma 2 (BCL2) [14–17] inhibitor of apoptosis, among others [18]. Each class of drug yields extended remissions with variable time periods of treatment ranging from intermittent to continuous. Sequential treatment with these agents is also effective. However, a subset of patients develop resistance to inhibitors of BCR pathways and BCL2 and have a relatively poor prognosis, spotlighting the need for further investigation of novel therapies [19]. Preventing this from occurring in high risk patients is also important for which new therapies are needed. Moreover, approximately 2–10% of CLL cases will progress to develop a highly aggressive lymphoma morphologically resembling a diffuse large B cell lymphoma (DLBCL) termed Richter’s Transformation (RT). Patients who develop RT have a poor prognosis with short survival due to the lack of effective therapies [20, 21].

Investigating mechanisms behind the genetic and clinical heterogeneity of CLL, genomic studies have identified a number of recurrent somatic mutations affecting several critical regulatory proteins, including, among others, those affecting the nuclear export protein Exportin-1 (XPO1; also known as Chromosome Region Maintenance 1, CRM1) [22–24]. Those affecting XPO1 are primarily observed as single point mutations to a residue within the NES-binding groove (most frequently E571K or E571G), altering the charge and structural basis of the amino acid in this critical location [25]. These recurrent hot-spot point mutations have been reported in ~ 5% of CLL cases and between ~ 25 and 35% of CLL cases progressing to RT, suggesting either a pathogenic role for this genetic aberration or association with other high risk features [24, 26–28].

**Fig. 1:**
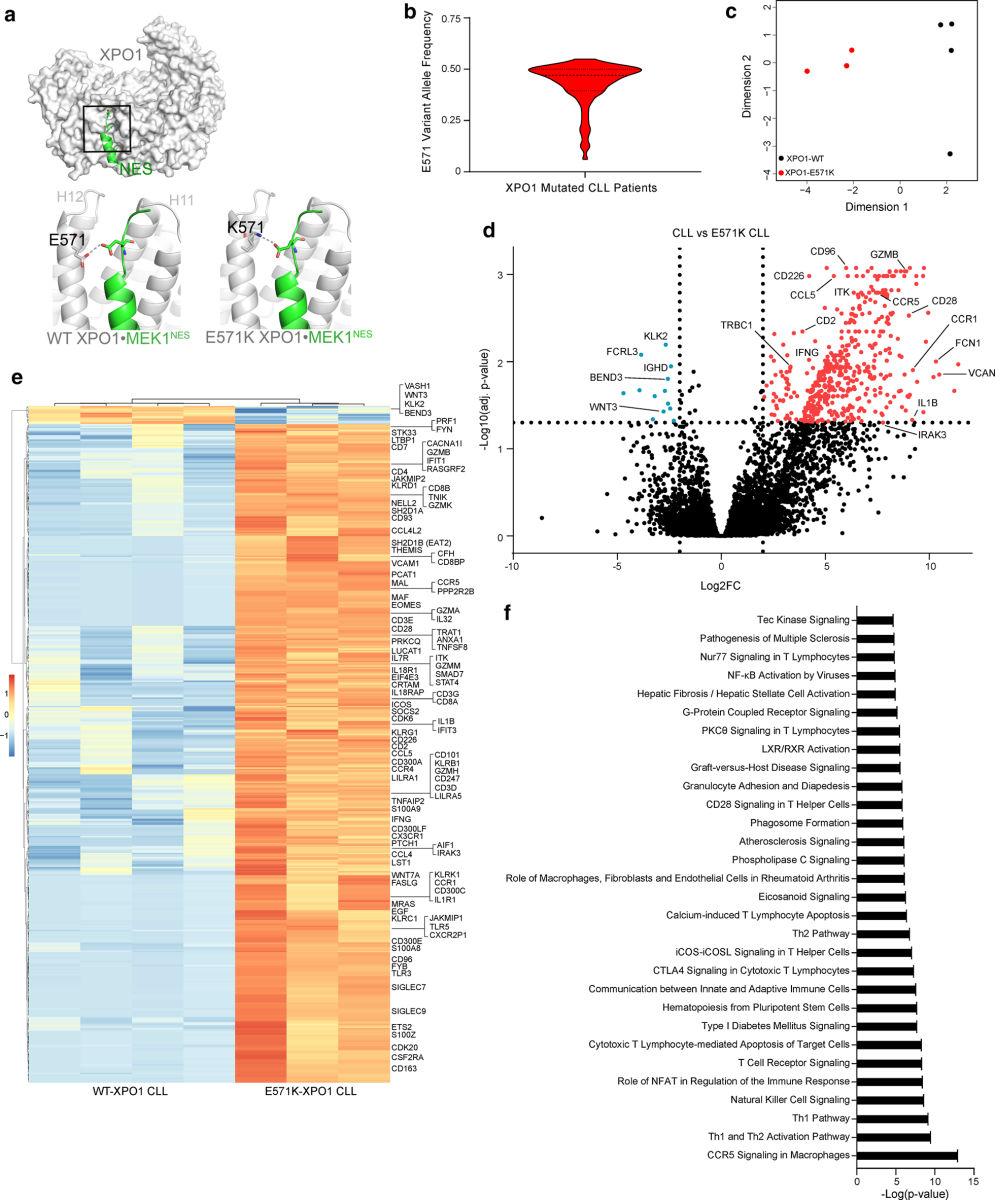
E571 XPO1 mutations occur in CLL and disrupt the transcriptome of the CLL cell. Structure of wildtype XPO1 (grey surface; RanGTP and RanBP1 in the complex are not shown for clarity) with an NES peptide (green cartoon) bound in NES-binding groove, which is located on the convex side of the protein between HEAT repeats H11 and H12. Details of the boxed area is shown in the bottom left panel: the NES peptide, Mek1^NES^, binds close to (3.4 Å) the side chain of E571 of wildtype XPO1. Bottom right: the E571K mutation places a lysine at position 571, which makes electrostatic interactions with a glutamate side chain of Mek1^NES^ causing the NES to bind much tighter to the XPO1 mutant [50]. Due to its simplicity, interactions with the E571G mutation are not shown. **a** Violin plot depicting the variant allele frequency (VAF) in CLL patients with an E571 XPO1 mutation. A median VAF of 0.47 (25th percentile = 0.39, 75th percentile = 0.50) suggests the heterozygous E571 XPO1 mutation is present in the dominant clone in these patients. Fewer than 5 XPO1-mutated CLL cases presented a VAF < 0.20. **b** Multidimensional scaling (MDS) plot of the top 1000 most variable genes in CLL cells isolated from patients with (E571K; *n* = 3) and without (WT; *n* = 4) an E571K XPO1 mutation demonstrate distinct clustering between mutated and non-mutated CLL samples along MDS dimension 1. **c** Volcano plot depicting adjusted p-values (FDR) versus log twofold change between E571K XPO1 and WT XPO1 IGHV-U CLL samples. Differentially expressed genes were selected with a fold change cutoff of 4 at 5% FDR. Significant genes enriched in E571K-mutated CLL cell samples are shown in red, and significant enrichment of genes in wt-XPO1 CLL cell samples are shown in blue. Along with upregulation of known supportive oncogenes, E571K XPO1 mutated CLL samples appear to demonstrate an increase in T cell-specific genes. **d** Heat map demonstrating differential expression of genes (|FC|> 4, FDR < 0.05) between E571K XPO1 (*n* = 3) and WT XPO1 (*n* = 4) IGHV-U CLL samples. Genes of interest are labeled. **e** Ingenuity Pathway Analysis (IPA) software analysis using significant differentially expressed genes in E571K XPO1 mutated CLL samples reveals perturbed downstream pathways enriched in T cell-related signaling and proliferation pathways. Enriched pathways are sorted by –Log(*p* value)

Nuclear vs cytoplasmic compartmentalization is a defining trait of eukaryotic cells, and remains critical for proper cell maintenance and function. Selective permeability of the nuclear envelope and the nuclear pore complex (NPC) allows for passive diffusion of small ions and metabolites while facilitating active transport of large macromolecules (> 40 kDa) with energy-dependent nuclear transporters. The karyopherin-β superfamily of importins and exportins mediates the transport of large macromolecules containing either nuclear localization signals (NLSs) or nuclear export signals (NESs), respectively [29]. The major nuclear export receptor, XPO1, exports hundreds of functionally diverse proteins, binding to highly diverse 10–15 amino acid NES motifs in cargos that contain 4–5 hydrophobic residues that interact with the hydrophobic XPO1 NES-binding groove (Fig. 1a) [30–33]. XPO1 is expressed in all human cells and has been cited as a core essential gene, where loss of XPO1 is a lethal event due to the ablation of proper nuclear and cytosolic organization [34, 35].

While maintaining proper homeostatic balance in eukaryotic cells, XPO1 sustains relevance to cancer through several proposed mechanisms; most widely cited is its ability to shuttle tumor-suppressor proteins out of the nucleus (e.g. p53, IkB, BRCA1) thereby preventing their anti-apoptotic function [36]. In addition, XPO1 is a transporter for > 1000 putative protein cargos [37–39], influences the localization and metabolism of oncoprotein-encoding mRNAs and various RNA species (miRNA, rRNA, snRNA, tRNA) [40], and plays an essential role in regulating mitosis and chromosome structure [41], suggesting many contributing oncogenic mechanisms may involve XPO1. Moreover, alterations to NESs of recurrently mutated genes in cancer have been shown to drive mis-localization of the NES-containing protein and enhance downstream tumorigenic effects [42]. In this regard, XPO1 is frequently overexpressed and/or present with recurrent mutations in many cancer types. Surprisingly, these abnormalities have remarkable specificity for hematologic malignancies including Hodgkin’s lymphoma (HL), DLBCL, primary mediastinal B cell lymphoma (PMBCL), and CLL [24, 43]. Notably, the *XPO1* gene is located on chromosome 2p15, suggesting *XPO1* overexpression may be a critical leukemogenic mechanism in the subset of CLL patients with gain of the short arm of chromosome 2 (+ 2p) [44].

Due to the over-expression of *XPO1* in many cancer cells and its role in exporting tumor suppressor genes such as TP53, development of selective inhibitors of nuclear export (SINEs) that inhibit the nuclear export activity of XPO1 in malignant cells has garnered increased attention [43, 45]. The SINE molecules selinexor (KPT-330/XPOVIO™) [46] or Eltanexor (KPT-8602) [47] have each demonstrated anti-leukemia activity in pre-clinical studies and have advanced to clinical trials in patients with CLL and other types of cancer. Moreover, Selinexor has received accelerated approval from the US Food and Drug Administration (FDA) for treatment of patients with relapsed and/or refractory (R/R) multiple myeloma or R/R DLBCL [48, 49].

A previous report described studies indicating that germline insertion of the E571K *XPO1* mutation could accelerate development of lymphoma in mice made to express high-level c-Myc and BCL-2, suggesting such XPO1 mutations could contribute to oncogenic transformation [24]. Additionally, we previously showed that the E571K mutation affects the binding of a small subset of NESs that have highly charged β-strands located close enough to make strong interactions with residue 571 [50], suggesting a select group of cargos are affected by this mutation. One of these cargos is the RNA regulator eIF4E-Transporter (4E-T), which forms a weaker association with E571K XPO1, resulting in inappropriate 4E-T localization in cells carrying the mutation. However, these previous studies employed mouse models used to study lymphomagenesis or models primarily for general in vitro tumor cell investigation. Although the E571K mutation in *XPO1* has been observed in CLL, the role they play driving leukemogenesis remains unclear.

Herein, we present a retrospective analysis of CLL cases in which we have identified a subset of patients harboring an E571 *XPO1* mutation. This analysis reveals the E571K/E571G *XPO1* mutation associated with high-risk genetic and epigenetic co-factors and at the time of diagnosis. To elucidate whether such mutations in XPO1 could play a causal role in CLL leukemogenesis, we developed Eµ-XPO1 transgenic mice harboring human *XPO1* under control of µ heavy chain enhancer (Eµ) elements driving constitutive expression of *XPO1*. We found that overexpression of *XPO1* (wt, E571K or E571G) was sufficient to initiate development of a CLL-like disease in a subset of aged mice, but lacked the ability to drive a spontaneous leukemic expansion in young mice. However, when crossed with the established Eµ-TCL1 CLL-mouse model (Eµ-XPO1/TCL1), concurrent presence of E571K or E571G *XPO1* mutations with overexpression of the *TCL1* oncogene sufficiently enhanced the rate in which these mice developed their CLL-like phenotype. Lastly, crystal structures of XPO1 (wt or E571K) bound to several SINE molecules (KPT-185, KPT-330/Selinexor, and KPT-8602/Eltanexor) show the compounds binding similarly in each case, which was further confirmed by a similar cytotoxicity profile from this class of compounds in patients’ cells with or without an XPO1 mutation. This suggests XPO1 inhibitors are likely to remain effective pharmaceutic candidates for cancer patients with E571 *XPO1* mutations.

## Methods

### CLL patient cohort selection

CLL patient information was collected from 4 independent cohorts as previously described [51]. In brief, a total of 1286 samples were collected, those from The Mayo Clinic (Mayo; *n* = 248) contributing treatment-naïve patients at diagnosis; samples collected from MD Anderson Cancer Center (MDACC; *n* = 367) were from treatment-naïve patients who subsequently required treatment; samples collected from The Ohio State University (OSU; *n* = 232) were from relapsed/refractory (R/R) patients (median 3 prior therapies) receiving single-agent ibrutinib in clinical trials conducted at OSU; and samples collected from the CLL Research Consortium (CRC; *n* = 439) were samples collected from treatment-naïve patients. All blood samples were obtained after patients provided written informed consent in accordance with the Declaration of Helsinki and established Institutional Review Board guidelines.

### CLL sample acquisition

Human de-identified CLL and normal B cells were isolated and cultured as previously described. Blood from newly diagnosed untreated CLL patients were obtained from the Ohio State University Leukemia Tissue Bank after obtaining informed consent approved by the cancer institution review board according to the Declaration of Helsinki. IgVH mutation status and epigenetic maturation status of patient CLL cells was determined via protocols previously described [52]. Proliferation of CLL cells was determined via MTS assay. Cells in all MTS assays were seeded at 5e^5^ cells/well in a 96-well plate for 48hrs with or without SINE treatment.

### Library construction and RNA-seq

Library preparation and RNA-sequencing was performed and analyzed under protocols developed and approved by the Genomic Services Laboratory at Nationwide Children’s Hospital. Downstream pathway analysis of differentially expressed genes was determined via publically available Ingenuity pathway analysis software as previously described [53]. Paired-end 150 bp reads were aligned to the human reference genome GRCh38 using HISAT2 [54] and gene counts were generated with featureCounts [55] as described in [56]. Gene expression was quantified as log2 counts per million and differential expression analysis was performed using R limma voom function [57]. Heatmaps were generated using R pheatmap package.

Mouse IGH gene usage was determined from RNA-sequencing. Cell pellets were collected and washed in PBS on ice prior to resuspension in TRIzol reagent and stored at − 80°. B-cells were isolated via EasySep Mouse Pan-B Cell Isolation Kit (Stemcell Technologies) from spleen suspensions. RNA was extracted using the Monarch Total RNA Miniprep Kit (New England Biolabs) according to manufacturer instructions. Total RNA was quantified using the Nanodrop 2000 Spectrophotometer (Thermo Fisher Scientific). The Clontech SMARTer v4 kit (Takara Bio USA, Inc.) was used for global preamplification. Illumina sequencing libraries were derived from the resultant cDNA using the Illumina Nextera XT DNA Library Prep Kit following manufacturer's instructions. Libraries were sequenced using an Illumina HiSeq 4000 sequencer paired-end 150 bp protocol to approximately 12 million passed filter clusters per sample. Data processing was performed according to the CLEAR workflow [58], which identifies reliably quantifiable transcripts in low-input RNA-seq for differentially expressed gene (DEG) transcripts using gene coverage profiles. BCR reads were binned by specific heavy (H) and light-chain V genes. First, we quantified the percentage of reads with heavy, kappa, and lambda chains compared to the number of total sequencing reads. MiXCR [59] (v3.0.5) was used with default parameters to identify preprocessed reads containing CDR3 regions from B-cell heavy, kappa, and lambda chains, generating a list of unique CDR3 sequences associated with their relative abundances and specific V(D)J gene usage.

### Animal studies

All animals were housed at The Ohio State University Comprehensive Cancer Center (OSUCCC). All animal experiments were carried out under protocols approved by the Ohio State University Institutional Laboratory Animal Care and Use Committee. Homozygous Eµ-TCL1 transgenic mice (background strain b6) have been previously described [60]. Eµ-XPO1 mice (E571WT, E571G, E571K; background strain C57BL/6) were generated via pronuclear injection of the pBH plasmid containing full-length hXPO1 cDNA into mouse embryos at the single-cell stage in collaboration with the Ohio State University Genetically Engineered Mouse Modeling Core (GEMMC). Embryos were implanted into pseudo pregnant females. Detection of hXPO1 mRNA in Eµ-XPO1 progeny was confirmed via RT-qPCR with human-specific probes (ThermoFisher, #Hs01034172_m1) and overexpression of XPO1 protein in Eµ-XPO1 progeny was confirmed via western blot (Santa Cruz, #sc-5595). Eµ-XPO1 mice and Eµ-TCL1 mice were crossed to produce Eµ-XPO1/Eµ-TCL1 progeny (abbreviated Eµ-XPO1/TCL1). Genotypes of the resulting progeny were confirmed via PCR. Whole blood from all transgenic mice was collected via submandibular vein (cheek punch) and assessed for leukemic development via flow cytometry. Pre-defined euthanasia criteria for mice in all transgenic colonies and murine transplant models included lethargy, impaired motility, splenomegaly, enlarged lymph nodes, and decrease in body weight (> 20%). Organs from select mice were collected at time of euthanasia and preserved in 10% formalin for histopathologic analysis. Complete blood count (CBC) analysis of whole blood was performed and analyzed under protocols developed and approved by The Ohio State University Comparative Pathology & Mouse Phenotyping Shared Resource.

### Flow cytometry

Fresh cells were isolated from spleen, thymus, bone marrow, or whole blood from mice at ages indicated. T and B cells were enriched using EasySep kit (Stem Cell Technologies, Vancouver, BC, Canada). Flow optimizations and protocols were used as previously described [61]. Gating strategy and graphs from representative C57BL/6 and Eµ-TCL1 whole blood are shown in the supplementary materials. All gates were set using fluorescent antibody controls prepared for each individual experiment. Flow cytometry antibodies used in this study include: anti-mouse CD45 (BD #559,864), anti-mouse CD5 (BD #553,023), anti-mouse CD19 (BD #562,701), anti-mouse CD45R/B220 (BD #553,088), anti-human CD3 (BD #555,335), anti-human CD19 (BD #562,440), anti-human CD2 (BD #555,326), anti-human CD28 (BD #555,729), anti-human Granzyme B (BD #561,142), anti-human IFN-gamma (BD #554,551). All data were analyzed using Kaluza v2.0 software (Becton Dickinson).

### Histopathology

Organs were harvested from colony mice at indicated ages or upon meeting euthanasia criteria. Tissues were fixed, processed and stained with hematoxylin and eosin (H&E) (Leica) as previously described [61]. Photographs were taken using an Olympus SC30 camera with an Olympus BX53 microscope. Peripheral blood smears were fixed and stained according to Wright-Giemsa stain standard procedures (Fisherbrand Hema3), and imaged with a Revolve R4 brightfield microscope, Echo Laboratories (model: RVL-100-G).

### Structure determination of XPO1-KPT complexes

E571K XPO1 (represented by ScXPO1 E582K) was cloned by quick-change mutagenesis of the E582 codon. The previously published pGexTev-ScCRM1* (contains residues 1–1058, Δ377–413, 537-DLTVK-541 to GLCEQ) crystallization construct for wildtype (WT) XPO1 was used as the PCR template for the E571K XPO1 mutant protein [33]. For structure determination of WT XPO1 bound to KPT-330, a construct of pGexTev-ScCRM1* was used where residues 537-DLTVK-541 were not mutated. The XPO1 proteins were expressed in E. coli (DE3) cells with 0.5 mM IPTG at 25 °C for 12 h. XPO1 was purified from lysates in GF buffer (20 mM HEPES pH 7.5, 100 mM NaCl, 5 mM MgOAc, and 2 mM DTT, and protease inhibitors) by affinity chromatography using Glutathione Sepharose 4B beads (GE Healthcare Life Sciences, PA), cleaved with TEV protease, and further purified using size exclusion chromatography (HiLoad 16/600 Superdex 200 pg, GE Healthcare Life Sciences). Yrb1 (Sc RanBP1(62–201)) was expressed from a pGEX-TEV construct, in E. coli (DE3) cells (0.5 mM IPTG for 12 h at 25 °C) and purified from lysates using the same purification scheme as for XPO1. A pET-15b-human Ran was used to express Ran (same expression condition as for Yrb1), lysed in in buffer containing 50 mM HEPES (pH 8.0), 2 mM magnesium acetate, 200 mM NaCl, 10% (vol/vol) glycerol, 5 mM imidazole (pH 7.8), 2 mM DTT, purified using Ni–NTA Agarose (Qiagen, Hilden, Germany), loaded with GMPPNP as previously described [33], and then further purified by anion exchange and size exclusion chromatography in TB buffer (20 mM HEPES pH 7.5, 110 mM KOAc, 2 mM MgOAc, 10% glycerol, and 2 mM DTT).

KPT-185, -330 (Selinexor), and -8602 (Eltanexor) were supplied by Karyopharm Therapeutics, LLC. The structure of wildtype XPO1 construct used for structure determination bound to KPT-330 does not contain the 537-DLTVK-541 to GLCEQ mutations present in the other XPO1 constructs. All the other wildtype XPO1 constructs contain the 537-DLTVK-541 to GLCEQ mutations that served to mimic the human XPO1 NES-binding groove. KPT-CRM1-Ran-RanBP1 complexes were assembled in a 3:1:3:3 molar ratio and purified by gel filtration using Superdex 75 10/300 GL (GE Healthcare Life Sciences), concentrated to 10 mg/ml, crystallized at 20 °C in 17% (weight/vol) PEG3350, 100 mM Bis–Tris (pH 6.4), 200 mM ammonium nitrate, and 10 mM Spermine HCl and crystals cryoprotected with the same crystallization condition supplemented with up to 23% PEG3350 and 12% glycerol followed by flash freezing in liquid nitrogen. X-ray diffraction datasets were collected at the APS 19ID beamline at the Argonne National Laboratory and processed using HKL-3000 [62]. Structures were determined by molecular replacement using the unliganded CRM1-Ran-RanBP1 structure (PDB 4HB2) as search model in PHENIX. Reiterative modeling in Coot and refinement were performed using Phenix until satisfactory MolProbity statistical measures were achieved [63, 64].

### Statistical analyses

Analyses were performed using previously described models [43]. For all in vivo survival experiments, Kaplan–Meier estimates of the survival function were calculated for each group and differences assessed using the log-rank test. In Fig. 3c, multiple founder lines (*n* = 5) were generated within each transgenic mouse genotype and were combined for analysis. Therefore, a shared frailty model was applied to the data in order to account for correlations among mice from the same founder; models included sex as a covariate. All survival analysis were performed using SAS/STAT software (SAS v9.4 for Windows, SAS Institute, Inc., Cary, NC); data were plotted using GraphPad Prism 8.4.2.

## Results

### Recurrent *XPO1* mutations E571K and E571G in CLL associate with poor prognosis

Human XPO1 is a ~ 120 kDa protein comprised of 21 repetitive anti-parallel α-helical segments forming tertiary protein structures termed ‘HEAT domains’ (H1-H21) [65–67]. The α-helices of H11 and H12 form a groove with hydrophobic pockets that serves as a binding site for prospective cargo proteins with an exposed hydrophobic NES (Fig. 1a). The E571 residue is located within this NES-binding pocket in a position close to the bound NES, where chemical and physical changes associated with mutations at this site (most often E571K or E571G) alter how XPO1 binds select NESs on these cargos [50] (Fig. 1a). While these mutations appear to be enriched in B cell malignancies, most notably in HL, DLBCL, PMBCL, or CLL [22–24], the effects that such mutations may have on the development or progression of these diseases are not understood.

To address this gap in understanding in CLL and to characterize the clinical significance of recurrent E571 *XPO1* mutations, we conducted a multi-center retrospective analysis on a large number of CLL cases (*N* = 1286), and identified 72 leukemia-cell samples (~ 6%) harboring a nonsynonymous mutation in *XPO1* at E571, resulting in an amino acid substitution of E→K in 59 (82%), E→G in 12 (17%), or E→A in 1 (1%) of the cases with *XPO1* mutations. The observed prevalence of each of these mutations at E571 was consistent with previous reports [22, 25, 68], and we define such mutations throughout the text as ‘mutated XPO1.’

In line with previous reports [35], the E571 variant allele frequency (VAF) present in *XPO1* mutated samples from the patient samples examined in this study had a high allelic frequency (median VAF = 0.47, 25% percentile = 0.39, 75% percentile = 0.50; Fig. 1b). Only four patient samples had mutated *XPO1* with a VAF < 0.2. One patient sample with biclonal disease had an E571K *XPO1* mutation with a VAF of 0.39 in the dominant CLL clone and an E571G *XPO1* mutation with a VAF of 0.10 in a smaller clonal population.

We interrogated the annotated clinical data to assess the relationship between *XPO1* mutations and patient outcome (Table 1). In addition to demographic (gender, age, Rai stage) and blood count information, (beta-2 microglobulin (B2M), hemoglobin (HGM), Lactate dehydrogenase (LDH), platelet count (PLT), and blood lymphocyte count (WBC)) routinely collected from CLL patients, we also obtained results from a genetic screening probing for significant cytogenetic abnormalities determined via fluorescence in situ hybridization (FISH) (del(11q22.3), del(13q14), del(17p13), trisomy 12 (+ 12), or cytogenetically normal (CN)) and gene mutation signatures with known leukemogenic potential (*NOTCH1*, *SF3B1*, *TP53*, *XPO1*, *EGR2*, *MYD88*). These characteristics were then stratified by *IgHV* mutation status or by epigenetic maturation status, which previous studies defined as major prognostic factors in CLL [51, 69, 70].

**Table 1:** Multi-Cohort CLL Patient Characteristics by IgHV status*

		All CLL patients^†^ (% or 95% CI)	XPO1-mut (IgHV-U)^†^ (% or 95% CI)	XPO1-wt (IgHV-U)^†^ (% or 95% CI)	XPO1-wt (IgHV-M)^†^ (% or 95% CI)	*p* value^‡^	test	
Demographics	Gender (*n*, Male)	649 (69.3%)	41 (75.9%)	349 (71.8%)	258 (65.2%)	0.630	χ2	
	Rai (*n*, II–IV)	167 (27.4%)	21 (53.8%)	86 (27.6%)	60 (23.3%)	0.199	χ2	
	Age (median, year)	57.2 (56.4–58.0)	52.3 (49.9–55.0)	57.1 (56.0–58.2)	58.7 (57.4–59.9)	0.031	t-test	
Blood Counts	B2M (median)	3 (2.8–3.2)	3.4 (2.6–4.2)	3.15 (2.9–3.4)	2.7 (2.5–2.9)	0.814	t-test	
	HGB (median)	13.1 (12.9–13.3)	12.5 (11.7–13.3)	13.1 (12.8–13.4)	13.4 (13.1–13.7)	0.562	t-test	
	LDH (median)	493 (463–523)	528 (446–610)	499.5 (453–546)	473 (438–508)	0.809	t-test	
	PLT (median)	167 (159.7–174.3)	140.5 (113.5–167.5)	177 (188–166)	164.5 (154.7–174.3)	0.149	t-test	
	WBC (median)	65.35 (57.67–73.03)	122.95 (82.28–163.62)	72.3 (61.83–83.60)	44.8 (35.59–54.01)	0.041	t-test	
FISH	del(11q)	74 (15%)	3 (11%)	63 (25%)	8 (4%)	0.081	χ2	
	del(13q)	276 (55%)	20 (74%)	116 (45%)	140 (66%)	0.003	χ2	
	del(17p)	51 (8%)	1 (3%)	40 (12%)	10 (3%)	0.079	χ2	
	12 +	64 (13%)	0 (0%)	42 (16%)	22 (10%)	0.011	χ2	
	CN	134 (27%)	7 (26%)	71 (28%)	56 (27%)	0.51	χ2	
Mutation Panel	NOTCH1	99 (9%)	9 (13%)	79 (13%)	11 (3%)	0.52	χ2	
	SF3B1	52 (15%)	8 (30%)	29 (14%)	15 (12%)	0.045	χ2	
	TP53	97 (19%)	5 (16%)	71 (26%)	21 (10%)	0.141	χ2	
	XPO1	72 (6%)	72 (100%)	0 (0%)	0 (0%)	-	-	
	EGR2	46 (4%)	4 (6%)	32 (5%)	10 (2%)	0.51	χ2	
	MYD88	26 (2%)	0 (0%)	2 (0.3%)	24 (6%)	0.81	χ2	

		All CLL patients^†^ (% or 95% CI)	XPO1-mut (LP-CLL)^†^ (% or 95% CI)	XPO1-wt (LP-CLL)^†^ (% or 95% CI)	XPO1-wt (IP-CLL)^†^ (% or 95% CI)	XPO1-wt (HP-CLL)^†^ (% or 95% CI)	*p* value^$^	Test
	Multi-Cohort CLL patient characteristics by epigenetic maturation status*							
Demographics	Gender (count, M)	659 (69%)	41 (78.8%)	310 (70.9%)	120 (72.7%)	188 (62.5%)	0.130	χ2
	Rai (count, > 1)	167 (27.5%)	21 (56.8%)	76 (27%)	36 (35.3%)	34 (18.2%)	0.036	χ2
	Age (median, year)	57.2 (56.4–58.0)	52.3 (49.6–55.0)	57.0 (55.8–58.1)	57.1 (55.2–59.1)	59.1 (57.7–60.6)	0.020	t-test
Blood Counts	B2M (median)	3 (2.8–3.2)	3.4 (2.6–4.2)	3.3 (3.0–3.6)	2.9 (2.5–3.3)	2.65 (2.4–2.9)	0.826	t-test
	HGB (median)	13.1 (12.9–13.3)	12.1 (11.3–12.9)	13 (12.7–13.3)	13.3 (12.8–13.8)	13.4 (13.1–13.7)	0.440	t-test
	LDH (median)	494 (464–524)	545 (459–631)	524 (475–573)	476 (415–537)	459 (422–496)	0.936	t-test
	PLT (median)	167 (160–174)	132 (105–159)	176 (165–187)	160 (145–175)	167 (155–179)	0.110	t-test
	WBC (median)	65.35 (57.67–73.03)	124 (81.34–166.66)	77.3 (65.49–89.11)	42.45 (26.59–58.31)	43.65 (33.02–54.28)	0.056	t-test
FISH	del(11q)	74 (14%)	2 (8%)	62 (26%)	9 (10%)	1 (1%)	0.064	χ2
	del(13q)	288 (56%)	18 (69%)	101 (42%)	64 (72%)	105 (64%)	0.0043	χ2
	del(17p)	52 (7%)	1 (3%)	39 (13%)	5 (4%)	7 (3%)	0.196	χ2
	12 +	66 (13%)	0 (0%)	43 (18%)	10 (11%)	13 (8%)	0.006	χ2
	CN	140 (27%)	8 (31%)	67 (29%)	18 (20%)	47 (29%)	0.54	χ2
Mutation Panel	NOTCH1	105 (9%)	11 (17%)	75 (13%)	11 (6%)	8 (3%)	0.289	χ2
	SF3B1	57 (15%)	7 (25%)	28 (15%)	18 (32%)	4 (4%)	0.131	χ2
	TP53	101 (19%)	5 (16%)	68 (28%)	13 (14%)	15 (9%)	0.214	χ2
	XPO1	71 (6%)	69 (97%)	0 (0%)	0 (0%)	0 (0%)	–	–
	EGR2	48 (4%)	5 (7%)	29 (5%)	9 (5%)	5 (2%)	0.32	χ2
	MYD88	27 (2%)	0 (0%)	0 (0%)	14 (7%)	13 (4)	–	–

^*^Characteristics at time of diagnosis

^†^Represents the total count of eligible patients with available data for each annotation

^‡^*p* value determined by: XPO1-mut (IgVH-U) vs XPO1-wt (IgVH-U)

^$^*p* value determined by: XPO1-mut (LP-CLL) vs XPO1-wt (LP-CLL)

As has been previously reported, CLL samples with mutated *XPO1* invariably were found to use unmutated *IgHV* (72/72 cases, 100%; IgHV-U) in contrast to samples with wt *XPO1*, for which only 633 of the 1,068 evaluable cases (59%) used unmutated *IgHV* (Additional file 1: Fig. S1A). Cases with *XPO1* mutations also had a skewed epigenetic maturation status. Sixty-nine of the 71 evaluated leukemia-cell samples (97%) had a low-programmed (LP-CLL) epigenetic signature [51]; in contrast, only 578 of the 1100 cases (53%) without detectable *XPO1* mutations had such an epigenetic signature. These data confirm previous reports that associated E571 *XPO1* mutations in CLL with these adverse prognostic markers [71].

As virtually all samples harboring mutated *XPO1* had either unmutated *IgHV* or LP-CLL, we next examined this data for clinical features associated with mutated *XPO1* that could distinguish such patients from patients with CLL cells that did not have *XPO1* mutations but using unmutated *IgHV* or present an LP-CLL epigenetic signature (Table 1). We found that patients with CLL cells harboring mutated *XPO1* were diagnosed at a significantly younger median age, and more often presented with an advanced Rai stage than patients with CLL that expressed unmutated *IgHV* or had LP-CLL signatures but had wt *XPO1*. Patients with CLL cells that had mutated *XPO1* also had higher absolute blood lymphocyte counts (WBC) at time of diagnosis than comparable patients with CLL cells that had wt *XPO1*.

Deletions in the long arm of chromosome 13 at q14 (del(13q14)) are observed in approximately 50% of all CLL cases by FISH analysis, but only in 45% or 42% of cases with unmutated *IgHV* or LP-CLL epigenetic signatures, respectively, as observed in our cohort of CLL samples (Table 1). However, cases with mutated *XPO1* for which we had cytogenetic data were more commonly associated with del(13q14) than were wt *XPO1* samples with unmutated *IgHV* (74% vs 45%; *p* = 0.003) or LP-CLL (69% vs 42%; *p* = 0.004). Furthermore, cases with mutated *XPO1* more commonly had nonsynonymous mutations in *SF3B1* than samples with wt *XPO1* and unmutated *IgHV* (30% vs 14%, *p* = 0.045) (Table 1). On the other hand, whereas nearly 16–18% of samples with wt *XPO1* and unmutated *IgHV* or LP-CLL had trisomy 12 (+ 12) at diagnosis, none of the samples with mutated *XPO1* had this chromosomal abnormality (*p* = 0.011, *p* = 0.006, respectively).

Despite having an advanced stage and younger age at diagnosis, patients with CLL cells harboring *XPO1* mutations did not display a strong difference in median survival than CLL patients that had wt *XPO1* and unmutated *IgHV* or LP-CLL (Additional file 1: figure S1B). Likewise, the difference in time to first treatment (TTFT) for patients with mutated *XPO1* relative to that of patients with wt *XPO1* and unmutated *IgHV* or LP-CLL also did not reach statistical significance (Additional file 1: figure S1C).

### CLL patients with the E571K mutation exhibit differential expression of genes important in the pathogenesis of CLL

We hypothesized that CLL cells containing an E571 *XPO1* mutation may become reliant upon alternative growth and proliferation signals stemming from disrupted nuclear transport of intracellular cargos, priming the B lymphocytes for enhanced oncogenic potential. Thus, we aimed to identify disrupted cellular pathways in response to the E571K *XPO1* mutation, the mutation most frequently cited in CLL patients. To do this, we performed unbiased RNA-sequencing (RNA-seq) in CLL patient B-CLL cells containing an E571K *XPO1* mutation and compared this with *XPO1*-wt *IgVH*-*U* patient CLL cells. Multidimensional scaling (MDS) analysis of the top 1000 most variable genes demonstrated distinctly unique clustering along the first dimension for *XPO1*-E571K patient and *XPO1*-wt patient samples (Fig. 1c). This clustering pattern was driven by a number of genes that were either over- or under-expressed in *XPO1*-E571K patient cells (Fig. 1d), contributing to a unique gene expression pattern in patients with this mutation signature (Fig. 1e).

In particular, CLL cells with *XPO1*-E571K had enriched expression of several genes known to potentially contribute to lymphocyte activation (*i.e. IFNG*, *GZMB*, *CCR1*, *FCN1*, and *VCAN*). Significant downregulation of a select few genes were noted in CLL cells with *XPO1*-E571K compared to those with wt XPO1, including *KLK2*, *BEND3*, and *WNT3*. Interestingly, CLL cells with *XPO1*-E571K displayed significant upregulation of several genes expressed in activated T lymphocytes, including *ITK*, *CCL5*, *CCR5*, *CD2*, *CD28*, *CD96*, *CD226*, and *TRBC1*, among others. Enrichment in activation genes *CD2*, *CD28*, *GZMB*, and *IFNG* in *XPO1*-E571K CLL cells were also seen at the protein level as detected by flow cytometry (Additional file 1: Figure S1D). Ingenuity Pathway Analysis (IPA) of these differentially expressed genes in *XPO1*-E571K CLL cells confirmed an enrichment for T cell-specific signaling pathways, including T cell activation and regulation, autoimmunity, NFAT signaling, NF-κB activation, and Tec kinase signaling (Fig. 1f).

### XPO1 overexpression induces leukemogenesis in XPO1-transgenic mice

Despite implications as an independent oncogenic event in CLL patients, until recently, there have been no reports on whether *XPO1* mutations could induce a CLL-like disease. Taylor and colleagues have recently described a novel knock-in mouse model with heterozygous expression of *XPO1*-E571K in the murine B lymphocytes. Such mice did not develop a B cell malignancy without alloantigen stimulation, but when crossed with two lymphoma models (c-MYC and BCL2), the E571K mutation enhanced the lymphomagenic potential of c-MYC and BCL2, suggesting additional molecular abnormalities must be present for the E571K XPO1 mutation to incite an aggressive tumor progression [24].

**Fig. 2:**
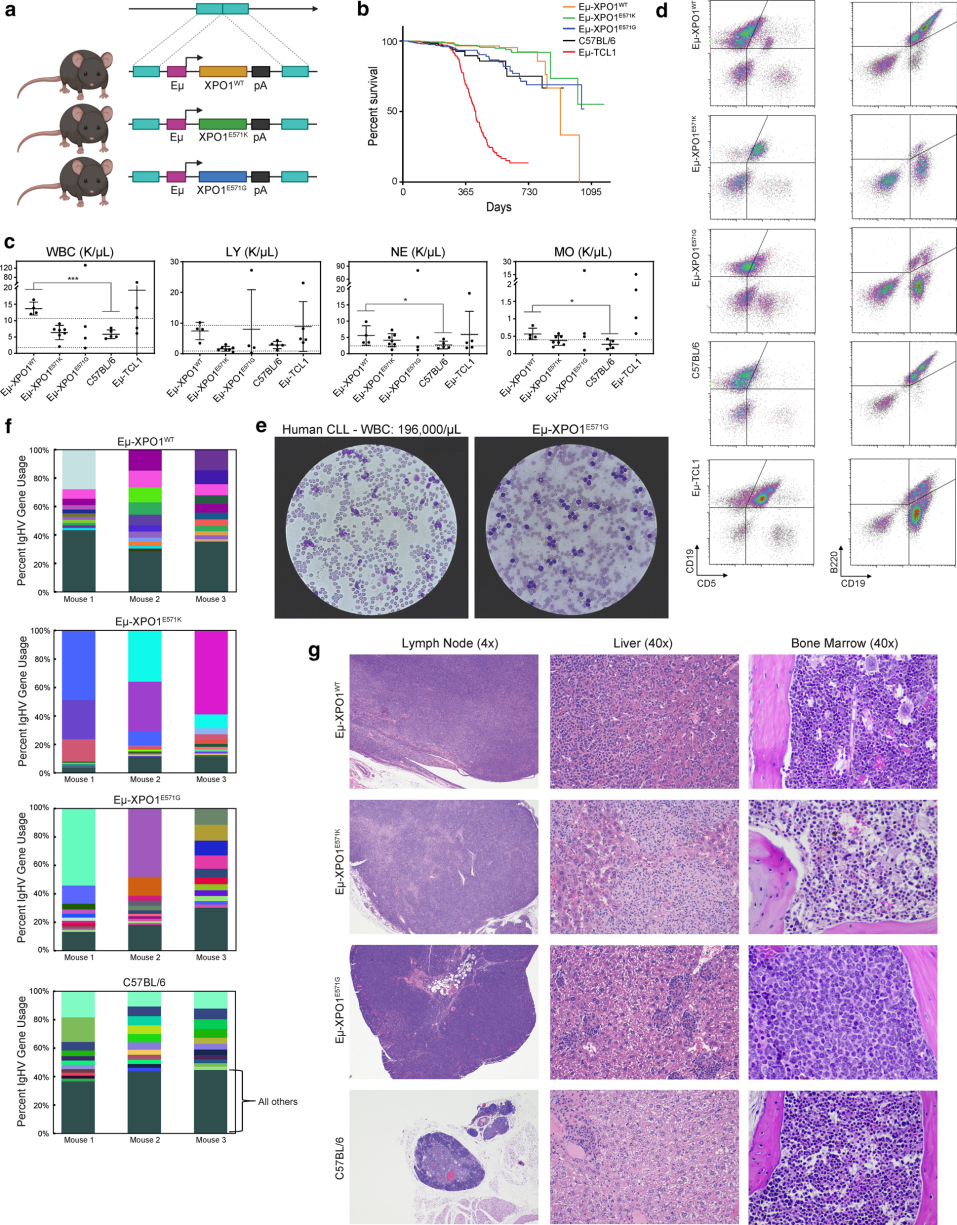
XPO1 abnormalities in murine B cells induce hematopoietic irregularities in significantly aged mice.** a** Schematic representing the Eμ-XPO1 transgenic mouse model, overexpressing either WT, E571K, or E571G XPO1 under immunoglobulin heavy chain (Eμ-) promoter/enhancer elements in c57bl/6 mice. For complete characterization, five founder lines were generated for each genotype. Artistic rendering created with Biorender.com. **b** A large cohort of Eμ-XPO1 transgenic mice were followed for overall survival (WT, *n* = 347; E571K, *n* = 578; E571G, *n* = 369) and compared with overall survival of C57BL/6 non-transgenic mice (*n* = 544) and Eμ-TCL1 transgenic mice (*n* = 246). Mice from all Eμ-XPO1 transgenic founder lines were combined for analysis. No significant changes in survival times between Eμ-XPO1 mice and C57BL/6 non-transgenic mice were observed, both extending past the median survival observed in Eμ-TCL1 mice. Statistical significance of Kaplan–Meier plot determined via log-rank (Mantel-Cox) test. **c** Automated CBC analysis of peripheral blood collected from aged Eμ-XPO1 mice (WT, *n* = 4; E571K, *n* = 7; E571G, *n* = 4) and age-matched C57BL/6 mice (*n* = 5). 12-month old Eμ-TCL1 mice (*n* = 5) were used for comparison. Mice from all Eμ-XPO1 groups were at an increased risk for elevated blood counts (in K/µL) compared to C57BL/6 mice, including total white blood cells (WBC), lymphocytes (LY), neutrophils (NE), and monocytes (MO). Dotted lines indicate the normal accepted range in each analysis. Bars represent mean ± SD. Missing bars indicate the SD exceeded the range determined by axis limits. Statistical significance determined via unpaired t test with Welch’s correction. **d** Immunophenotypic analysis of peripheral blood from aged (24–32 months) revealed an expansion of CD19 + /CD5 + or CD19 + /B22dim CLL-like cells in a subset of Eμ-XPO1 mice from each XPO1-WT, XPO1-E571K, and XPO1-E571G transgenic mice. This CLL-like expansion was similar to that commonly found in the Eμ-TCL1 mouse model. **e** Evaluation of a peripheral blood smear (40 × objective) from an Eμ-XPO1^E571^^G^ transgenic mouse reveals significant accumulation of lymphocytes in ratios similar to that of a human CLL patient with a blood count of 196. **f** Representative microscopic images from detailed histopathology analysis demonstrate a subset of aged Eμ-XPO1 mice display a higher incidence of hematopoietic cancer than non-transgenic mice. Seventy five percent (3/4) of analyzed mice from each group (XPO1-WT, XPO1-E571K, and XPO1-E571G) demonstrated hematopoietic neoplasia, consistent in morphology with histiocyte associated lymphoma. Neoplastic infiltrates were present in the livers and lymph nodes of all three mice, as well as the bone marrow (× 40) of a XPO1-E571G mouse. Lymph nodes (4x) and livers (40x) were significantly enlarged in Eμ-XPO1 due to accumulation of neoplastic lymphocytes. Most mice (3/4, 75%) from the C57BL/6 non-transgenic group showed no evidence of hematopoietic neoplasia. **g** Comparison of IGHV gene usage between splenic B cells from Eµ-XPO1 (XPO1-WT, XPO1-E571K, and XPO1-E571G) and C57BL/6 non-transgenic mice (*n* = 3 per group). Eµ-XPO1^E571K^ and Eµ-XPO1^E571G^ mice demonstrated a majority of BCR usage among the top 10 IGH genes, suggesting less diversity in the BCR repertoire compared to non-transgenic mice. Usage of BCR reads were binned by heavy chain V gene names, with each V gene normalized by the total number of heavy-chain reads. The top 10 most abundant genes are shown in triplicate mice with shared V genes maintaining the same color across all triplicates and samples. Genes not in the top 10 in terms of usage were grouped as “others” (bottom-most segment in each stacked-bar) and shown as a dark forest-green color (see label). Gene names of the top 10 V genes in order of abundance are presented in Additional file 4: Table S1

In an alternate strategy, we developed transgenic mice harboring wt *XPO1* or mutant *XPO1* under the control of the VH-promoter-IgH-Eμ-enhancer elements to generate Eµ-XPO1^WT^, Eµ-XPO1^E571K^, Eµ-XPO1^E571G^ transgenic mice, which had high level expression of the *XPO1* transgene in mature B cells (Fig. 2a, Additional file 2: Figure S2A-C) [60]. Five unique founder lines were generated for each Eµ-XPO1^WT^, Eµ-XPO1^E571K^, Eµ-XPO1^E571G^ transgenic strain. No outstanding phenotypic differences were observed between founder lines of mice expressing *XPO1*-wt, *XPO1*-E571K, or *XPO1*-E571G, respectively (data not shown), and founder lines were combined for comparative analysis.

Immunophenotypic evaluation of mice in Eμ-XPO1 colonies by flow cytometry revealed no overt change in B and T cell subsets in the spleen or blood of mice between 3 and 12 months of age (Additional file 2: Fig. S2D–G). This included no change in transitional B cells in the spleen or circulating B cells in the blood, and no changes in B cells populating the follicular (FOL) or marginal zones (MZ/MZP) of the spleen in 12 month old animals. In 3 month old animals, no marked changes were observed in CD4 + or CD8 + T cell subpopulations between Eμ-XPO1 and C56BL/6 non-transgenic mice. Within T cell subsets, no strong differences in naïve, memory, or effector compartments were observed. And further, both CD4 + and CD8 + T cell subsets displayed comparable percentages of Ctla4, Pd-1, Cd25, or Tim3 expressing cells. Large scale longitudinal evaluation of Eμ-XPO1 transgenic colonies resulted in similar overall survival between Eμ-XPO1 and C57BL/6 non-transgenic colonies, with both Eμ-XPO1 colony and C57BL/6 non-transgenic survival times extending significantly past the median survival observed in Eμ-TCL1 CLL model (Fig. 2b).

Long term follow-up of mice advancing to 24–33 months of age, however, revealed Eμ-XPO1^WT^, Eμ-XPO1^E571K^ and Eμ-XPO1^E571G^ mice variably developed lymphocytosis and a CLL-like disease. Elevated circulating blood cell counts, including white blood cells (WBC), lymphocytes (LY), neutrophils (NE), and monocytes (MO), could be found moderately- or far-exceeding ranges found in C57BL/6 animals, most notably in the Eμ-XPO1^WT^ group (Fig. 2c). Blood counts in Eμ-TCL1 mice variably exceeding those found in C57BL/6 mice are shown as reference. Furthermore, analysis of CD45 + cells circulating in the blood (Additional file 2: Figure S2H) demonstrated that a small subset (5/15 analyzed mice) of aged Eμ-XPO1^WT^, Eμ-XPO1^E571G^, and Eμ-XPO1^E571K^ mice spontaneously developed a circulating leukemia characterized by expansion of CD5^+^/CD19^+^ and CD19^+^/B220dim B lymphocytes (Fig. 2d). These neoplastic cells resembled the CLL-like disease observed in Eμ-TCL1 transgenic animals. Evaluation of blood mononuclear cells collected from one Eμ-XPO1^E571G^ animal revealed significant accumulation of well-differentiated lymphocytes in ratios resembling the blood smear of a CLL patient with a WBC count of 196,000/µL (Fig. 2e).

Using RNA-sequencing methods previously described by our group [72], we identified *IgHV* transcript use in aged Eμ-XPO1 mice (range 19–28 months). BCR reads from the variable (V) regions of each heavy- (H) and light-chain genes were binned and normalized by the total number of reads corresponding to each respective chain. Percent *IgHV* gene usage in Eμ-XPO1 and C57BL/6 non-transgenic mice are visualized in Fig. 2f, with each color bar representing the 10 individual *IGHV* genes with the highest usage, and the dark green bar representing the collection of all other *IGHV* genes found in that animal. We found that Eμ-XPO1^E571G^ or Eμ-XPO1^E571K^ mice that did not have overt CLL-like lymphocytosis had splenic B cells expressing a restricted *IgHV* repertoire, suggesting nascent leukemogenesis (Additional file 4: Table S1). This trend was not observed in the non-transgenic animals, where a high degree of IGH gene diversity was observed. Of note, a less restrictive use of *IgHV* genes in aged Eμ-XPO1^WT^ mice relative to that of age-matched Eμ-XPO1^E571G^ or Eμ-XPO1^E571K^ mice.

To assess the disease burden accumulated throughout all tissues, aged Eμ-XPO1^WT^, Eμ-XPO1^E571G^, and Eμ-XPO1^E571K^ mice were sacrificed and detailed necropsy was performed. Microscopic examination of tissue architecture revealed development of a systemic hematopoietic neoplasia most commonly affecting the liver and lymph nodes in 75% (3/4 mice per group) of the analyzed Eμ-XPO1 transgenic mice (Fig. 2g, Additional file 2: Figure S2I). Livers and lymph nodes from Eµ-XPO1^WT^, Eµ-XPO1^E571G^ and Eµ-XPO1^E571K^ mice were significantly enlarged due to infiltration of neoplastic cells composed (in varying ratios) of large lymphocytes and histiocytoid cells. Accumulation of neoplastic infiltrates was also observed in the bone marrow of a Eµ-XPO1^E571G^ mouse. This neoplasm was most consistent with histiocyte associated lymphoma, a variant of diffuse large B-cell lymphoma and homologue of histiocyte and T-cell rich DLBCL in humans [73]. The spleen, kidney, and lungs were also affected in analyzed mice although at a lesser frequency. Additional lesions were noted in these very old mice without obvious differences between groups, including glomerulopathies, chronic kidney injury, aggregates of mixed lymphocytes in various tissues, extramedullary hematopoiesis, lymphoid atrophy, and occasional neoplasms of non-lymphoid tissue. In comparison, the aged-matched C57BL/6 non-transgenic group showed some evidence of hematopoietic neoplasia in 25% (1/4 mice) of the analyzed animals.

### E571 XPO1 mutations accelerate leukemogenesis in Eµ-TCL1 transgenic mice

**Fig. 3:**
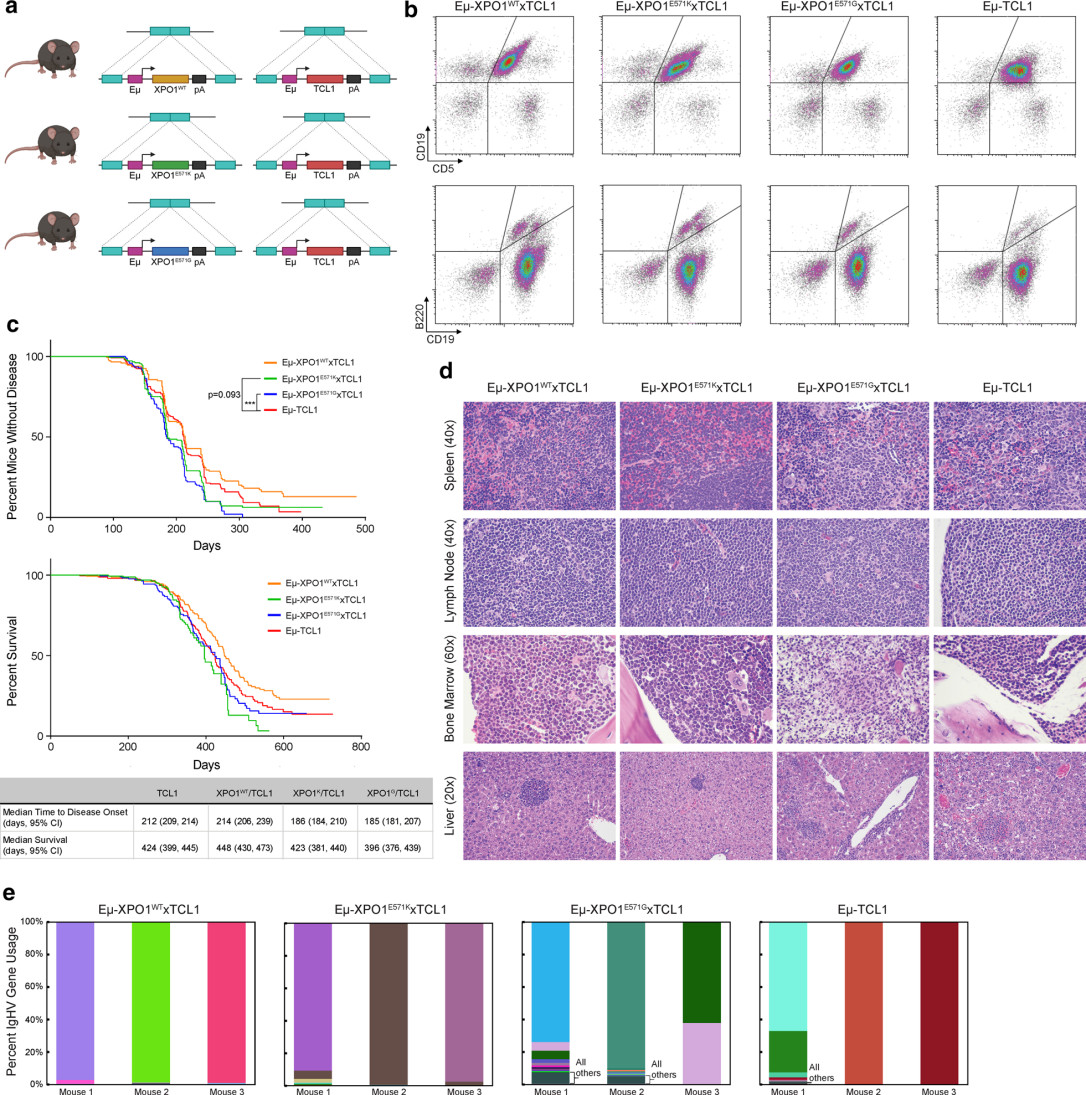
E571K and E571G XPO1 mutations accelerate disease onset in the Eµ-TCL1 mouse model. **a** Schematic representing the Eμ-XPO1xTCL1 double transgenic mouse model, overexpressing either WT-, E571K-, or E571G-XPO1 under immunoglobulin heavy chain (Eμ-) promoter/enhancer elements in c57bl/6 mice. **b** Eμ-XPO1xTCL1 and Eμ-TCL1 mice were followed monthly by flow cytometry analysis for development of a CLL-like disease circulating in the peripheral blood. Mice from each Eμ-XPO1^WT/E571K/E571^^G^xTCL1 colonies spontaneous developed an aggressive CD19 + /CD5 + and CD19 + /B22dim CLL-like disease similar to that observed in the Eμ-TCL1 model. **c** Eμ-XPO1xTCL1 and Eμ-TCL1 mice were followed monthly by flow cytometry analysis for development of a CLL-like disease circulating in the peripheral blood, and the time to disease onset (> 20% CD19 + /CD5 + and CD19 + /B22dim population in peripheral blood) was recorded for each mouse. Mice from Eμ-XPO1^E571K^xTCL1 (*n* = 188) and Eμ-XPO1^E571G^xTCL1 (*n* = 174) colonies displayed an accelerated time to disease onset compared to mice in the Eμ-TCL1 (*n* = 247) model (*p* = 0.093, *p* = 0.001, respectively). No change in rate of disease onset was observed between Eμ-XPO1^WT^xTCL1 (*n* = 151) and Eμ-TCL1 mice (p = 0.713). Regardless of disease status, mice were continuously monitored for emergence of symptoms qualified as reaching early removal criteria (ERC), recording the survival time for each mouse. No significant difference in overall survival was noted between Eμ-XPO1xTCL1 (WT, *n* = 151, E571K, *n* = 189; E571G, *n* = 173) and Eμ-TCL1 mice (n = 246). A slight survival advantage was observed in Eμ-XPO1^WT^xTCL1 mice compared to Eμ-TCL1 mice, although no level of significance was reached (*p* = 0.331). Statistical significance of Kaplan–Meier plots were determined via log-rank (Mantel-Cox) test. *p < 0.05. **p < 0.01. ***p < 0.001. **d** Comparative histopathology from Eμ-XPO1xTCL1 and Eμ-TCL1 transgenic mice. All mice examined developed lymphoid neoplasia, similar in character to the Eµ-TCL1 transgenic mouse model. Neoplastic lymphocytes were widely disseminated, often affecting the spleen (× 40), lymph nodes (× 40), bone marrow (× 60), and liver (× 20). **e** Comparison of IGHV gene usage between splenic B cells from Eµ-XPO1xTCL1 (XPO1-WT, XPO1-E571K, and XPO1-E571G) and Eμ-TCL1 mice (n = 3 per group). All mice demonstrated remarkably low diversity in BCR usage, suggesting a distinct clonal expansion comprises the tumor burden in these animals. Usage of BCR reads were binned by heavy chain V gene names, with each V gene normalized by the total number of heavy-chain reads. The top 10 most abundant genes are shown in triplicate mice with shared V genes maintaining the same color across all triplicates and samples. Genes not in the top 10 in terms of usage were grouped as “others,” and, if present, are shown as the bottom most segment in a dark forest-green color (see label). Gene names of the top 10 V genes in order of abundance are presented in Additional file 4: Table S1

We crossed mice from each respective Eµ-XPO1 transgenic line with Eµ-TCL1 mice, a model known to develop an overt B cell leukemia between 7 and 12 months of age [60, 74, 75] (Eµ-XPO1xTCL1; Fig. 3a). Eµ-XPO1xTCL1 double transgenic mice were monitored monthly for onset of leukemia by flow cytometry analysis of blood mononuclear cells. Immunophenotypic profiling demonstrated a similar expansion of detectable CD5^+^/CD19^+^ and CD19^+^/B220dim cells in the blood of Eµ-TCL1 and all Eµ-XPO1xTCL1 mice over time (Fig. 3c). However, by monitoring the time to disease onset (determined as > 20% CD5^+^/CD19^+^ and CD19^+^/B220dim populations), we observed accelerated leukemic development in Eµ-XPO1^E571G^xTCL1 and Eµ-XPO1^E571K^xTCL1 mice when compared to Eµ-TCL1 mice (median time to disease onset = 185 vs 212 days, *p* = 0.001; 186 vs 212 days, *p* = 0.093, respectively; Fig. 3c). Alternatively, overexpression of WT-XPO1 in Eµ-TCL1 B lymphocytes did not enhance the rate of leukemia onset when compared to Eµ-TCL1 mice (median time to disease onset = 214 vs 212 days, *p* = 0.713).

Despite accelerated time to disease onset, we observed no marked changes in overall survival between Eµ-XPO1^E571G^xTCL1 or Eµ-XPO1^E571K^xTCL1 mice and Eµ-TCL1 transgenic mice (Median survival = 396 vs 424 days, *p* = 0.254; 423 vs 424 days, *p* = 0.360, respectively; Fig. 3c).

Histopathologic examination between age-matched (7–16 months) Eµ-XPO1xTCL1 transgenic mice (*n* = 6, respectively) revealed that mice in all groups demonstrated a histologic disease phenotype similar to the Eµ-TCL1 mouse (Fig. 3d). Mice developed lymphoid neoplasia affecting multiple organ systems, including thymus, spleen, lymph node, bone marrow, liver, kidney, and gastrointestinal tract, consisting of small to large lymphocytes mixed with varying proportions of histiocytes. This type of murine neoplasm has been previously identified in the Eµ-TCL1 mouse [76], warranting a diagnosis of histiocyte-associated lymphoma.

Again assessing the BCR repertoire in transgenic animals by evaluating IGH transcripts of splenic B cells by RNA-sequencing, we observed distinct usage of select few *IGHV* gene transcripts in all Eµ-XPO1xTCL1 groups and in all Eµ-TCL1 mice, suggesting these spleens are comprised of malignant B lymphocytes with remarkably low clonal diversity (Fig. 3e, Additional file 4: Table 1).

### E571K XPO1 mutation does not alter SINE therapeutic potential

Recent advances in therapeutic strategies for management of hematologic malignancies have identified SINE molecules having potential anti-cancer activity. First generation (KPT-185, KPT-330, KPT-335) and second generation SINE therapeutics (KPT-8602) (Additional file 3: Figure S3A) have demonstrated selective cytotoxicity for solid and liquid tumor types in pre-clinical and clinical studies. Such activity is due to slowly-reversible covalent binding with the C528 residue within the XPO1 NES-binding groove, effectively inhibiting nuclear transport of essential proteins and RNAs [43, 77–79]. One SINE compound, Selinexor (KPT-330/XPOVIO™), was recently approved by the FDA for use in therapy of patients with in penta-refractory multiple myeloma or R/R DLBCL [45, 49, 80].

**Fig. 4:**
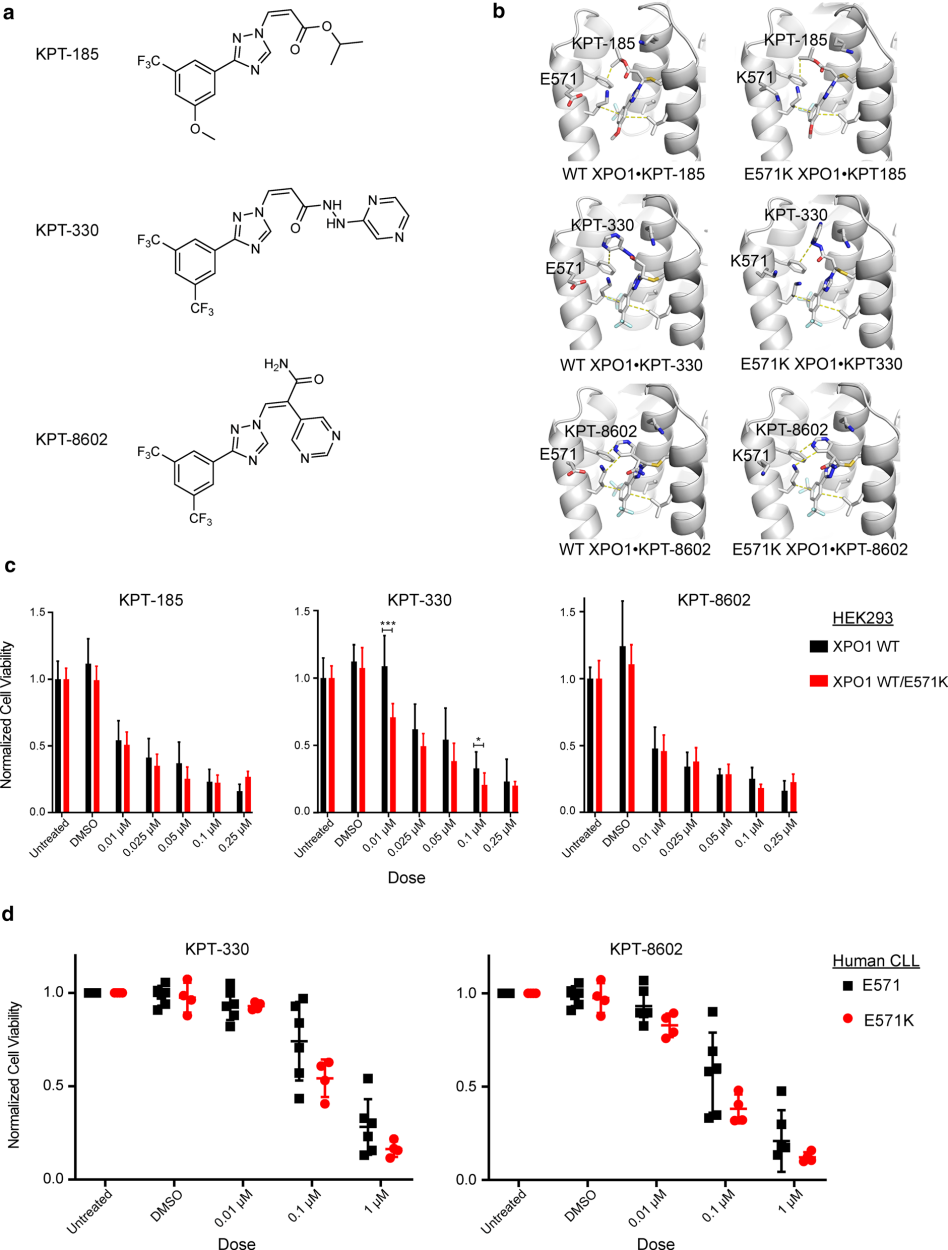
E571K XPO1 mutation does not disrupt the biophysical mechanism of action for candidate SINE therapeutics.** a** 2-dimensional chemical structure of SINE molecules KPT-185 (top), KPT-330 (selinexor; middle), and KPT-8602 (eltanexor; bottom). **b** 3-dimensional rendering of crystal structures for wildtype- (WT) and E571K-XPO1 bound to SINE molecules KPT-185 (top), KPT-330 (selinexor; middle), and KPT-8602 (eltanexor; bottom). Select XPO1-KPT interactions are shown with dashed lines. Electron density for these structures are shown in the supplemental information. No significant changes to drug-target interactions are observed between wt- and E571K-XPO1. **c** Proliferation potential of wt or heterozygous XPO1-E571K expressing HEK293 cells upon exposure to SINE molecules KPT-185 (left), KPT-330 (selinexor; middle), and KPT-8602 (eltanexor; right) determined via MTS assay after 48 h. A similar dose-dependent response was observed between both wt and E571K-XPO1 expressing HEK293 cells with each SINE molecule. Proliferation was normalized to the untreated condition. Statistical significance was determined using the Two-stage linear step-up procedure of Benjamini, Krieger and Yekutieli, with Q = 1%. “*” is indicative of *p* < 0.05, and “***” is indicative of *p* < 0.001. **d** Proliferation potential of wt or heterozygous XPO1-E571K expressing human CLL cells upon exposure to clinically relevant SINE molecules KPT-330 (selinexor; left) and KPT-8602 (eltanexor; right) determined via MTS assay after 48 h. A similar dose-dependent response was observed between both wt and E571K-XPO1 expressing CLL cells with each SINE molecule. Each point represents CLL cells from a different patient. Proliferation was normalized to the untreated condition. Statistical significance determined using the Holm-Sidak method, with alpha = 0.05. Each row was analyzed individually, without assuming a consistent SD

Glutamate 571 is located in the XPO1 NES-binding groove, and the E571K mutation changes both the shape and electrostatics of this exposed region [50] (Fig. 1a). The considerable change in size and charge resulting from this amino acid change may also affect SINE molecule binding within this groove. Reports exploring the relationship between SINE efficacy and tumor models with E571K *XPO1* have been discussed previously, suggesting a slight reduction in SINE efficacy in PMBCL cell lines (MedB1) [25] and an increase in SINE efficacy in CLL cell lines (NALM-6) [24]. However, as there has been no mechanistic study of the relationship between the E571K mutations and binding of the SINE compounds to XPO1, we solved the crystal structures for E571 (wt) and E571K XPO1 bound to SINE compounds KPT-185, KPT-330 or KPT-8602 (Table 2, Fig. 4a, b and Additional file 3: Figure S3B).

**Table 2:** Data collection and refinement statistics for XPO1 bound to KPT-SINEs

Crystals of XPO1•RanGTP•RanBP1 bound to KPT-SINEs						
*Data collection*						
XPO1	wildtype	wildtype	wildtype	E571K	E571K	E571K
KPT-SINE	KPT-185	KPT-330	KPT-8602	KPT-185	KPT-330	KPT-8602
Space group	P4_3_2_1_2					
Cell dimensions a = b, c (Å)	105.9, 305.5	105.4, 305.1	106.7, 306.1	106.0, 306.0	106.0, 305.6	106.0, 305.4
Resolution range (Å)	50.00–2.80 (2.85–2.80)	50.00–2.04 (2.10–2.04)	50.00–2.40 (2.44–2.40)	40.00–1.94 (1.97–1.94)	40.00–1.94 (1.97–1.94)	40.00–2.20 (2.24–2.20)
Multiplicity	15.1 (13.8)	4.4 (3.1)	16.6 (16.4)	17.6 (17.9)	17.2 (16.3)	15.8 (15.9)
Data completeness (%)	100 (100)	99.9 (99.2)	100 (100)	95.5 (91.1)	100 (100)	100 (100)
*R*_meas_ /*R*_pim_ (%)	18.5/5.6 (0.0/60.8)	11.5/6.3 (0.0/81.1)	14.0/5.6 (0.0/93.9)	9.2/2.1 (300.0/66.0)	10.6/2.5 (337.5/80.9)	15.7/4.0 (766.8/190.5)
I/σ(I)	12.7 (1.3)	10.4 (0.6)	19.7 (1.9)	38.2 (1.4)	35.6 (1.3)	25.5 (1.4)
CC_1/2_ (last resolution shell)^a^	0.656	0.096	0.576	0.486	0.477	0.709
*Refinement statistics*						
Resolution range (Å)	46.99–2.80 (2.87–2.80)	47.55–2.04 (2.09–2.04)	45.54–2.41 (2.46–2.40)	38.25–1.94 (1.99–1.94)	38.20–1.94 (1.97–1.94)	38.17–2.19 (2.23–2.19)
No. of reflections *R*_work_/R_free_	3181/185 (798/47)	6191/350 (5532/276)	5191/159 (3579/109)	9650/169 (3610/63)	12,080/206 (2704/46)	7562/176 (2595/61)
Data completeness (%)	83.1 (28)	98.3 (80.15)	97.2 (76)	89.9 (40)	67.7 (22)	79.7 (35)
Atoms (non-H protein and ligand/solvent)	10,807/0	11,851/616	11,102/0	11,093/1147	11,103/511	11,102/444
*R*_work_/*R*_free_ (%)	20.8/26.1 (28.0/35.8)	17.5/23.2 (38.1/43.0)	19.6/24.6 (24.2/31.4)	19.4/22.6 (26.6/26.0)	20.1/24.1 (30.4/34.9)	20.4/23.6 (33.3/31.9)
R.m.s.d. Bond length (Å)/angle (°)	0.009/1.039	0.004/0.872	0.014/1.214	0.003/0.550	0.004/0.144	0.003/0.521
Mean B-value (Å [2])	41.6	51.8	35.1	18.8	19.5	37
Ramachandran plot favored /disallowed (%)^b^	95.63/0.23	98.16/0.15	96.17/0.53	96.40/0.75	97.07/0.38	97.15/0.38
ML coordinate error	0.32	0.17	0.32	0.19	0.20	0.30
PDB code	6XJP	7L5E	6XJT	6XJR	6XJS	6XJU

Data for the outermost shell are given in parentheses

^a^Karplus PA & Diederichs K (2012) Linking crystallographic model and data quality. Science 336(6084):1030–1033

^b^As defined by MolProbity in PHENIX

The structures of KPT-bound E571 and E571K XPO1 were very similar. Comparison of wildtype and E571K XPO1 with all three sets of KPT compounds gave pairwise Cα r.m.s.d. values less than 0.18 Å and XPO1 side chains were in similar conformations, implying that both wt and E571K-XPO1 will confer similar XPO1-drug interactions (Fig. 4b). In all structures, there was no density for the side chain of Glu571 in wildtype XPO1 or for the lysine side chain of E571K XPO1, suggesting they are flexible and mobile in the crystal structure and unlikely to make any persistent contacts with the SINE compounds bound in this groove (Fig. 4b and Additional file 3: Figure S3B). Furthermore, while the variable side chains of the SINE compounds are likely flexible (weak to no density), the trifluoromethyl phenyl triazole scaffolds are well-defined and bind similarly to both wildtype and E571K XPO1 (Fig. 4 and Additional file 3: Figure S3A, B). All structural observations suggest the E571K mutation is unlikely to change how KPT-185, KPT-330 (Selinexor) or KPT-8602 bind to XPO1, and will have little to no effect on inhibition efficacy.

Furthermore, we evaluated the ability for these SINE molecules to impact cell proliferation using in vitro models expressing wildtype or E571K-mutated *XPO1*. First, employing a HEK293 tumor cell model we have previously designed for heterozygous expression of *XPO1*-E571K in the endogenous locus via CRISPR-based gene editing techniques [50], we observed a similar, dose-dependent, inhibitory response upon 48 h of exposure to SINE molecules KPT-185, KPT-330 or KPT-8602 between both wt and *XPO1*-E571K expressing cells (Fig. 4c). *XPO1*-E571K mutant HEK293 cells appeared to display an increase in sensitivity to treatment with KPT-330 (Selinexor) at 0.01 μM (p < 0.001) and 0.1 μM (p = 0.027) doses, however. A similar trend was observed when treating wt or *XPO1*-E571K expressing human CLL samples *ex-vivo* with clinically relevant SINE molecules KPT-330 and KPT-8602, observing no significant change in reduction of proliferative potential in *XPO1*-E571K expressing cells (Fig. 4d). While no level of significance was reached at these dosing schedules, however, a modest increase in sensitivity to SINE inhibition was again observed in *XPO1*-E571K expressing CLL cells. Collectively, these results suggest KPT-330 and KPT-8602 are likely to remain effective as a therapeutic strategy for CLL patients possessing an E571 *XPO1* mutation.

## Discussion

Wide scale genomic analysis across a range of cancer types have discovered the recurrent E571 *XPO1* mutations to be particularly enriched in hematologic malignancies. In CLL, presence of an E571 *XPO1* mutation also suggests association with other high-risk CLL prognostic indicators, but to date, a complete understanding of their impact on the genesis and progression of these hematologic malignancies remains unresolved. We have herein provided an extensive evaluation of the leukemogenic potential of E571 *XPO1* mutations in both human and murine CLL. Thanks to collaboration between a diverse set of clinical CLL research groups, we retrospectively analyzed a total of 1286 CLL cases, identifying 72 of which having presented with an E571 *XPO1* mutation at their time of diagnosis. The overall distribution of *XPO1*-mutated patients in this cohort and their association with other high-risk CLL co-factors falls in line with previous reports for this mutation, providing further evidence for the E571 *XPO1* mutation as a cancer-associated genetic abnormality. The high VAF seen among all patients suggests XPO1 is likely part of the early initiating event of CLL and is most likely not acquired as a later sub-clone with treatment. Collectively, this suggests a dominant role for XPO1 in CLL leukemia initiation. Despite these associations, the results of our study also confirm that the E571 *XPO1* mutation does not impact survival outcomes in these patients, measured either in surrogate by TTFT or from time from diagnosis to death.

Our study does highlight a handful of CLL patient characteristics found in those with an E571 *XPO1* mutation that may provide an insight to mechanisms of pathogenesis, however.

With regards to cytogenetic evaluation, poor survival outcomes paired with a significant association with del(13q14) and an abrupt dissociation with trisomy 12 (+ 12) in *XPO1*-mutated CLL patients offer potential pathways to explain the consequences of these mutations. Deletion of the q14 region of chromosome 13 in CLL cases is often met with a favorable prognosis, citing the loss of miR-15/16, DLEU7, or RB1 as major contributors to disease pathogenesis [81–83]. Roughly 70% of evaluable *XPO1*-mutated patients presented del(13q14) as a co-occurring genetic abnormality, a significant increase from the ~ 50% reported in the general CLL population and the ~ 42–45% reported in CLL cells with wt *XPO1*. Despite a historically fair prognosis, the survival outcomes of these patients were among the shortest reported in this study, suggesting some combination of dysfunctional molecular signaling stemming from these genetic aberrations are acutely advantageous for proliferation of the CLL cells. Moreover, the alternative activation of survival pathways in XPO1-mutated CLL cells suggests the XPO1 mutation may be skewing the molecular dependence of specific survival pathways for continued proliferation of neoplastic B cells and encourages the emergence of additional factors to contribute to leukemogenesis. Enrichment in factors such as *CD2*, *CD28*, *GZMB*, and *INFG* also suggests the disrupted signaling in *XPO1*-E571K CLL cells may influence surrounding immune cells, contributing to the immune dysfunction attributed to CLL patients. The role for complications stemming from E571 *XPO1* mutations in modulating the immune system of CLL patients, or increasing susceptibility to opportunistic infections [84], remain unresolved. Further genetic evaluation will be necessary to completely describe these mechanisms.

Of the most striking results from cytogenetic evaluation in these patients was the exclusivity of the E571 *XPO1* mutation and the gain in copy number for chromosome 12. This suggests a divergent biology of trisomy 12 and XPO1-initiated CLL. Copy number gains in entire chromosomes remain particularly challenging events to study in cancer pathogenesis due to the immensity of information stored in each chromosome. Regarding chromosome 12, however, a recent report suggests hyperactive NFAT signaling in + 12 CLL cases may act as a driving mechanism in these neoplastic cells [85]. Interestingly, RNA-sequencing of CLL cells with E571K *XPO1* mutations in our study revealed an upregulation of genes converging on NFAT and NFAT-related signaling pathways. This relationship may reveal a scenario in which convergence of the disrupted molecular pathways from both genetic aberrations is a lethal event in CLL cells. Thus, development of an E571 *XPO1* mutation would push towards an aggressive CLL phenotype with worse overall CLL prognosis, and conversely, development of a trisomy 12 leading to an intermediate CLL course.

It is important to note that the CLL patient data described in our study were collected from tertiary care centers and are likely selecting for advanced disease cases that are referred to these institutions. This remains a limitation to most large-scale investigations determining trends in cancer data, highlighting the need for widespread access to affordable, high-throughput evaluation of tumor samples in primary care centers.

Previous reports have noted that E571 *XPO1* mutations are exclusively found as heterozygous missense mutations, never appearing in the homozygous or hemizygous state [24, 86]. This finding reinforces the notion that XPO1 contributes critical homeostatic functions to the eukaryotic cell. As CLL is most often defined as a clonal expansion of malignant B lymphocytes, identifying a median E571 VAF in *XPO1*-mutated CLL patients to be 0.47 suggests the E571 *XPO1* mutation is in the dominant clone of the total disease burden. This finding proposes the E571 *XPO1* mutation as an early transformative event in progression of CLL, from which these clones progress to populate the majority of the leukemic tumor burden. This same explanation, however, implies the E571 *XPO1* mutation in B lymphocytes alone is not sufficient to spur development of an overt leukemia, as very few CLL patients presented with a low VAF at the time of diagnosis. This idea is reaffirmed by data generated from our novel Eμ-XPO1 transgenic mouse model, where E571K-XPO1 and E571G-XPO1 overexpressing mice displayed the same rates of disease development as mice overexpressing the WT-XPO1 transgene. However, splenic populations from Eμ-XPO1^E571K^ and Eμ-XPO1^E571G^ mice, but not Eμ-XPO1^WT^ mice, were largely comprised of clonally related B cells, suggesting these mutations are advantageous for proliferation and survival, and would be primed for leukemic transformation with the addition of another genetic abnormality.

When paired with another CLL-driving oncogene, like has been recently shown by Taylor and colleagues in mouse models combining the E571K *XPO1* mutation with c-Myc or Bcl-2 alterations [24], recurrent mutations to this site stimulate a more aggressive disease phenotype. In our study, crossing Eμ-XPO1 mice with the Eμ-TCL1 mouse, which is the most common murine model of CLL, Eμ-XPO1xTCL1 mice expressing E571K- or E571G-*XPO1* displayed a more rapid onset of their CLL-like disease. This accelerated disease phenotype was not produced in Eμ-XPO1xTCL1 mice expressing WT-XPO1. Interestingly, however, no significant impact on survival was observed between each Eμ-XPO1xTCL1 transgenic cross and Eμ-TCL1 mice. Moreover, the histologic disease phenotype in all Eμ-XPO1xTCL1 transgenic crosses retained similarity to that observed in the Eμ-TCL1 model, suggesting the overall phenotype resulting from this double transgenic cross is dominated by the TCL1 oncogene. Observations with this Eμ-XPO1xTCL1 mouse fall in line with survival data collected from human CLL patients, suggesting that once leukemogenic transformation is complete, other high risk factors may provide a more significant contribution to the overall mortality of this disease.

As the presence of an E571 *XPO1* mutation in CLL almost exclusively coincides with a negative overall prognosis, development of an effective therapeutic regimen is of critical importance. Recently, small molecule inhibitors of XPO1 termed SINEs have gained increasing attention as anti-cancer molecules, with specific emphasis toward hematologic malignancies. The covalent binding site of all SINE compounds, C528, resides in the XPO1 NES-binding groove within proximity of the E571 locus, the latter of which potentially contributing to the biochemical environment uniquely conducive for SINE binding. However, by solving crystal structures of SINE-bound XPO1, we determined the E571K mutation is unlikely to impact the inhibitory potential of this class of drugs. These data support the use of SINE molecules in cancer patients possessing an E571 XPO1 mutation without suspicion of alternative drug-target interactions altering the therapeutic potential. Our data using a human tumor cell model and CLL patient cells with heterozygous *XPO1*-E571K expression support this claim, as we observed no change in proliferation potential between wt and E571K mutant cells upon treatment with these molecules. In both mutant models, interestingly, a modest increase in sensitivity to SINE inhibition was observed at select doses, suggesting manipulation of molecular signaling pathways stemming from the E571 XPO1 mutation may make these cells more susceptible to this form of anti-cancer treatment. Further characterization of these interactions will be necessary to fully describe the altered molecular pathways stemming from E571 *XPO1* mutations that may influence the sensitivity and cytotoxic efficacy of tumor cells to inhibition by SINE molecules.

## Conclusion

The similar disease course in human and murine CLL observed in our study suggests the mislocalization of intracellular machinery stemming from E571-*XPO1* mutations may be priming pre-neoplastic B cells for complete leukemogenic transformation with the addition of oncogenic molecular defects converging on similar critical regulatory or signaling pathways. However, a complete understanding of the leukemogenic consequences stemming from the E571 *XPO1* mutation are not yet fully understood and will require further investigation to provide a complete description of this unique genetic abnormality.

## Supplementary Information

**Additional file 1:** **Figure S1**. E571 XPO1 mutations occur in CLL and are associated with high risk genetic and epigenetic markers. a Genetic screening at the time of diagnosis identified the IGHV mutation status and epigenetic maturation status of all CLL patients in our CLL cohort. CLL patients with an E571 XPO1 mutation almost exclusively associated with maintaining an un-mutated IGHV (IGHV-U) region and presenting with low-programmed CLL epigenetic markers (LP-CLL), each acting as markers for high-risk CLL. The distribution of XPO1 mutated patients with these risk factors significantly differs from the distribution seen in CLL cases with wt-XPO1. b Retrospective analysis of overall CLL patient survival as shown via Kaplan-Meier plot. Wt-XPO1 CLL patients were grouped by IGHV mutation status or epigenetic maturation status and plotted against XPO1-mutated CLL patients. Both XPO1-mutated CLL patients and wt-XPO1 (IGHV-U or LP-CLL) displayed significantly shorter survival times compared with CLL patients without these high-risk markers. No significant difference in survival between XPO1-mutated and wt-XPO1 (IGHV-U or LP-CLL) cases were observed. c Retrospective analysis of the time to first treatment (TTFT), a surrogate marker for CLL patient survival, as shown via Kaplan-Meier plot. Wt-XPO1 CLL patients were grouped by IGHV mutation status or epigenetic maturation status and plotted against XPO1-mutated CLL patients. Both XPO1-mutated CLL patients and wt-XPO1 (IGHV-U or LP-CLL) displayed significantly shorter TTFT compared with CLL patients without these high-risk markers. No significant difference in TTFT between XPO1-mutated and wt-XPO1 (IGHV-U or LP-CLL) cases were observed. Statistical significance of Kaplan-Meier plots were determined via log-rank (Mantel-Cox) test. *, p<0.05. **, P<0.01. ***, p<0.001. d E571K-XPO1 CLL patient samples display a higher frequency of CD19+CD2+, CD19+CD28+, CD19+GZMB+, and CD19+IFN-g+ B cell populations than in WT-XPO1 samples (n=7 per group) detected by flow cytometry. Plots represent cell populations after gating on CD19+ cells. GZMB and IFN-g expression were collected with and without 3 hour CpG stimulation. Statistical significance between groups determined via unpaired t-test with Welch’s correction. *, p<0.05. **, P<0.01.

**Additional file 2:** **Figure S2**. Establishment and characterization of the Eμ-XPO1 mouse model. a Founder lines for all three genotypes were established using human recombinant XPO1 DNA for overexpression of WT-XPO1, E571K-XPO1, or E571G-XPO1. Sanger sequencing of the recombinant expression vector confirmed the correct codon sequence for the E571 site (E517, GAA; E571K, AAA; E571G, GGA). b Expression of human XPO1 (hXPO1) mRNA in Eµ-XPO1 transgenic mice was confirmed via quantitative rt-PCR. hXPO1 mRNA was not detectable in C57BL/6 non-transgenic mice. Mouse TBP (mTBP) expression was used as a control. c Overexpression of XPO1 protein in Eµ-XPO1 transgenic mice was confirmed via western blot. Elevated presence of the XPO1 protein was noted in Eµ-XPO1 transgenic mice compared with C57BL/6 non-transgenic counterparts. Fold change (FC) value represents optical density quantification (XPO1/loading control). d Immunophenotypic analysis of B lymphocytes (Cd11b-/Cd3) populating the spleen and peripheral blood of 12-16 month old Eµ-XPO1 transgenic mice revealed no significant changes in percentage of transitional B lymphocytes (Cd93+/B220+) in either compartment compared with age-matched C57BL/6 non-transgenic counterparts (n=3 per group). e Immunophenotypic analysis of B lymphocytes (Cd19+/B220+/Cd5-) populating the spleen of XX month old Eµ-XPO1 transgenic mice revealed no significant changes in percentage of follicular (FOL; IgM+/Cd21dim) or marginal zone/marginal zone progenitor (MZ/MZP; IgM+/Cd21+) cells compared with C57BL/6 non-transgenic counterparts (n=3 per group). f Immunophenotypic analysis of T cell subsets revealed no significant changes in Cd3+, Cd4+, or Cd8+ populations between 3 month Eµ-XPO1 transgenic mice and age-matched C57BL/6 non-transgenic counterparts (n=3 per group). Within Cd4+ and Cd8+ T cell subsets, no significant alterations to naïve (Cd62L+/Cd44-), effector (Cd62L-/Cd44-), or memory (Cd62L+/Cd44+) T cell populations were observed. g Further immunophenotypic analysis of Cd4+ and Cd8+ T cell subsets in the peripheral blood revealed no significant alterations to Ctla-4, Pd1, Cd25, or Tim3 expression levels between age-matched Eµ-XPO1 transgenic mice and C57BL/6 non-transgenic counterparts (n=3 per group). h Gating strategy used to identify B lymphocytes from single Cd45+ cell populations circulating in the peripheral blood. i Representative comparative histopathology of enlarged lymph nodes (60x) from Eμ-XPO1 transgenic mice. Accumulation of neoplastic lymphocytes displaced normal tissue architecture as noted in the C57BL/6 mouse.

**Additional file 3:** **Figure S3**. Electron density of SINE-XPO1 structures. a Crystal structures of wildtype- (WT) and E571K XPO1 bound to KPT-185, KPT-330 (selinexor) and KPT-8602 (eltanexor) are shown with electron density (blue mesh) from composite omit maps contoured to 1.0 sigma.

**Additional file 4: Table S1**. IGH gene usage in Eμ-XPO1 and Eμ-XPO1xTCL1 mice.

## Data Availability

RNA-sequencing data for human CLL subjects can be found here: GSE163370. Crystallography data can be found here: PDB codes 6XJP, 7L5E, 6XJT, 6XJR, 6XJS, 6XJU. Other raw datasets analyzed during the current study are available from the corresponding author on reasonable request.

## Abbreviations

XPO1: Exportin 1
CRM1: Chromosome region maintenance 1
CLL: Chronic lymphocytic leukemia
BTK: Bruton’s tyrosine kinase
PI3K: Phosphoinositide 3-kinase
BCL2: B-cell lymphoma 2
DLBCL: Diffuse large B cell lymphoma
RT: Richter’s transformation
NPC: Nuclear pore complex
NES: Nuclear export signal
HL: Hodgkin’s Lymphoma
PMBCL: Primary mediastinal B cell lymphoma
R/R: Relapsed and refractory
MM: Multiple myeloma
4E-T: EIF4E-transporter
VAF: Variant allele frequency
B2M: Beta-2 microglobulin
HGM: Hemoglobin
LDH: Lactate dehydrogenase
PLT: Platelet
WBC: White blood cell
FISH: Florescence in Situ Hybridization
+ 12: Trisomy 12
CN: Cytogenetically normal
IgHV-U/M: IgHV-Unmuated/Mutated
LP-CLL: Low programmed epigenetic signature
IP-CLL: Intermediate programmed epigenetic signature
HP-CLL: High programmed epigenetic signature
TTFT: Time to first treatment
OS: Overall survival
MDS: Multidimensional scaling
FOL: Follicular zone
MZ/MZP: Marginal zone
LY: Lymphocytes
NE: Neutrophils
MO: Monocytes
BCR: B cell receptor
