# Supplementary figures and images for: Recurrent XPO1 mutations alter pathogenesis of chronic lymphocytic leukemia
Source: J Hematol Oncol. 2021 Jan 15;14:17. doi: 10.1186/s13045-021-01032-2 (PMC7809770; doi:10.1186/s13045-021-01032-2)

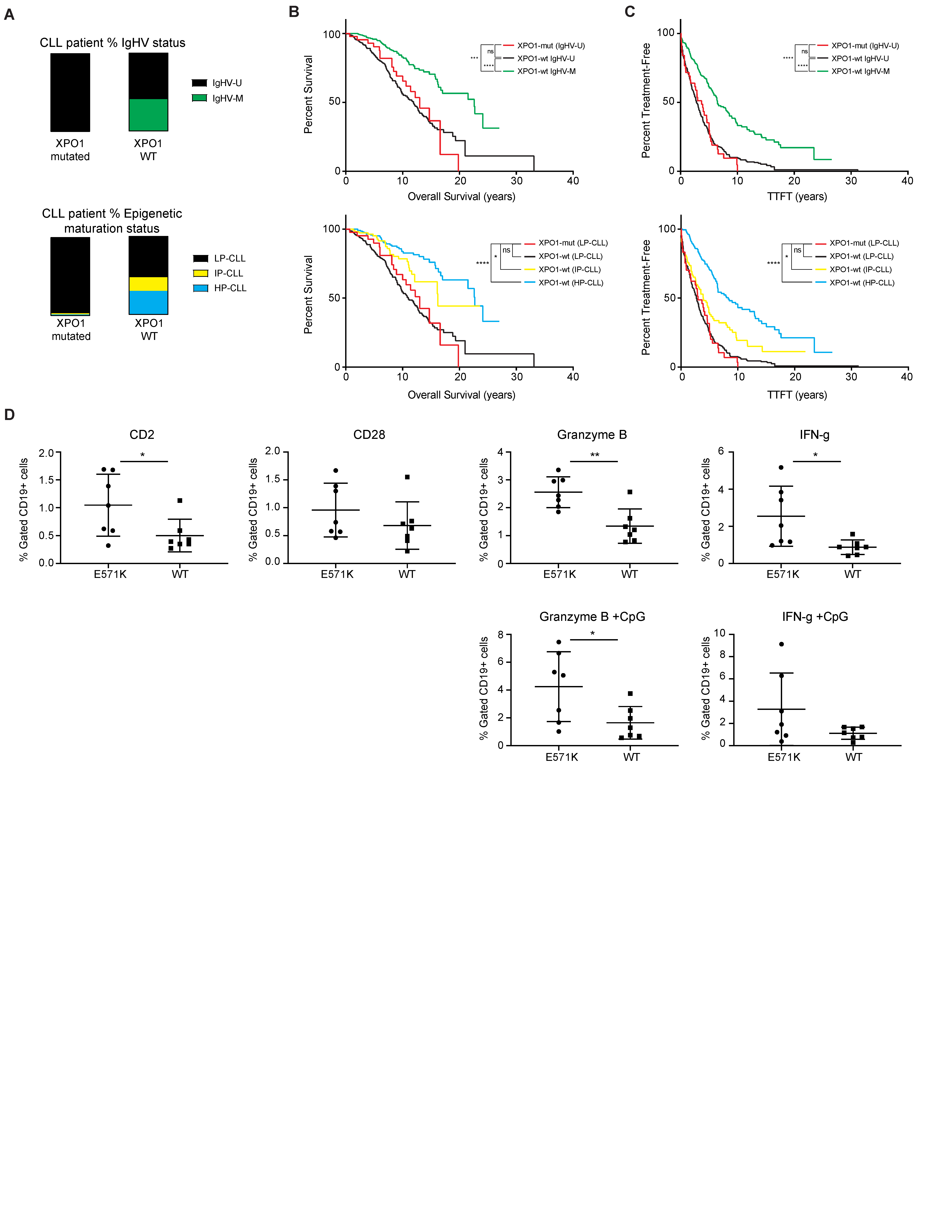

Supplement: Supplementary file 1 — Additional file 1: Figure S1. E571 XPO1 mutations occur in CLL and are associated with high risk genetic and epigenetic markers. a Genetic screening at the time of diagnosis identified the IGHV mutation status and epigenetic maturation status of all CLL patients in our CLL cohort. CLL patients with an E571 XPO1 mutation almost exclusively associated with maintaining an un-mutated IGHV (IGHV-U) region and presenting with low-programmed CLL epigenetic markers (LP-CLL), each acting as markers for high-risk CLL. The distribution of XPO1 mutated patients with these risk factors significantly differs from the distribution seen in CLL cases with wt-XPO1. b Retrospective analysis of overall CLL patient survival as shown via Kaplan-Meier plot. Wt-XPO1 CLL patients were grouped by IGHV mutation status or epigenetic maturation status and plotted against XPO1-mutated CLL patients. Both XPO1-mutated CLL patients and wt-XPO1 (IGHV-U or LP-CLL) displayed significantly shorter survival times compared with CLL patients without these high-risk markers. No significant difference in survival between XPO1-mutated and wt-XPO1 (IGHV-U or LP-CLL) cases were observed. c Retrospective analysis of the time to first treatment (TTFT), a surrogate marker for CLL patient survival, as shown via Kaplan-Meier plot. Wt-XPO1 CLL patients were grouped by IGHV mutation status or epigenetic maturation status and plotted against XPO1-mutated CLL patients. Both XPO1-mutated CLL patients and wt-XPO1 (IGHV-U or LP-CLL) displayed significantly shorter TTFT compared with CLL patients without these high-risk markers. No significant difference in TTFT between XPO1-mutated and wt-XPO1 (IGHV-U or LP-CLL) cases were observed. Statistical significance of Kaplan-Meier plots were determined via log-rank (Mantel-Cox) test. *, p<0.05. **, P<0.01. ***, p<0.001. d E571K-XPO1 CLL patient samples display a higher frequency of CD19+CD2+, CD19+CD28+, CD19+GZMB+, and CD19+IFN-g+ B cell populations than in WT-XPO1 samples (n=7 [file 13045_2021_1032_MOESM1_ESM.tif]

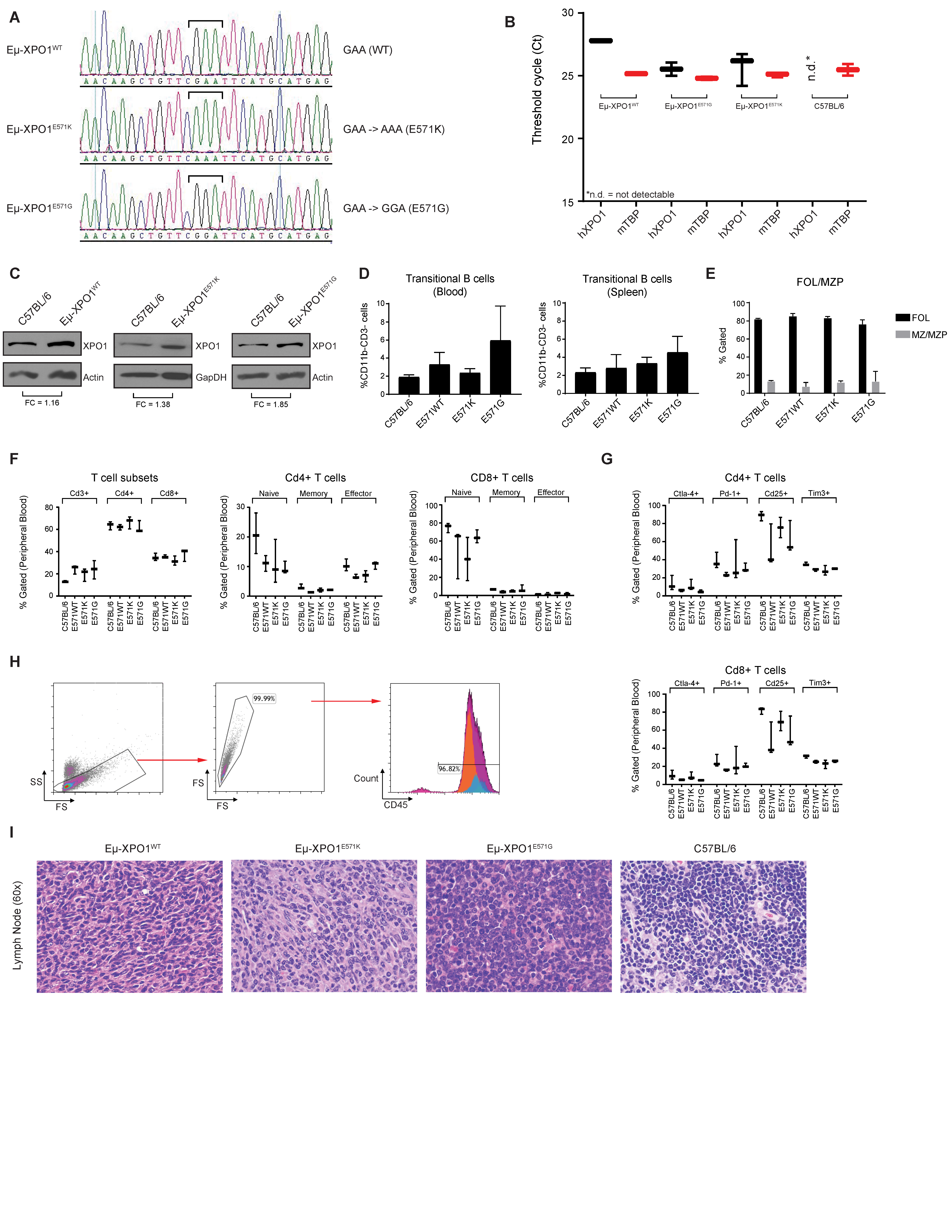

Supplement: Supplementary file 2 — Additional file 2: Figure S2. Establishment and characterization of the Eμ-XPO1 mouse model. a Founder lines for all three genotypes were established using human recombinant XPO1 DNA for overexpression of WT-XPO1, E571K-XPO1, or E571G-XPO1. Sanger sequencing of the recombinant expression vector confirmed the correct codon sequence for the E571 site (E517, GAA; E571K, AAA; E571G, GGA). b Expression of human XPO1 (hXPO1) mRNA in Eµ-XPO1 transgenic mice was confirmed via quantitative rt-PCR. hXPO1 mRNA was not detectable in C57BL/6 non-transgenic mice. Mouse TBP (mTBP) expression was used as a control. c Overexpression of XPO1 protein in Eµ-XPO1 transgenic mice was confirmed via western blot. Elevated presence of the XPO1 protein was noted in Eµ-XPO1 transgenic mice compared with C57BL/6 non-transgenic counterparts. Fold change (FC) value represents optical density quantification (XPO1/loading control). d Immunophenotypic analysis of B lymphocytes (Cd11b-/Cd3) populating the spleen and peripheral blood of 12-16 month old Eµ-XPO1 transgenic mice revealed no significant changes in percentage of transitional B lymphocytes (Cd93+/B220+) in either compartment compared with age-matched C57BL/6 non-transgenic counterparts (n=3 per group). e Immunophenotypic analysis of B lymphocytes (Cd19+/B220+/Cd5-) populating the spleen of XX month old Eµ-XPO1 transgenic mice revealed no significant changes in percentage of follicular (FOL; IgM+/Cd21dim) or marginal zone/marginal zone progenitor (MZ/MZP; IgM+/Cd21+) cells compared with C57BL/6 non-transgenic counterparts (n=3 per group). f Immunophenotypic analysis of T cell subsets revealed no significant changes in Cd3+, Cd4+, or Cd8+ populations between 3 month Eµ-XPO1 transgenic mice and age-matched C57BL/6 non-transgenic counterparts (n=3 per group). Within Cd4+ and Cd8+ T cell subsets, no significant alterations to naïve (Cd62L+/Cd44-), effector (Cd62L-/Cd44-), or memory (Cd62L+/Cd44+) T cell populations were observed. g Further immu [file 13045_2021_1032_MOESM2_ESM.tif]

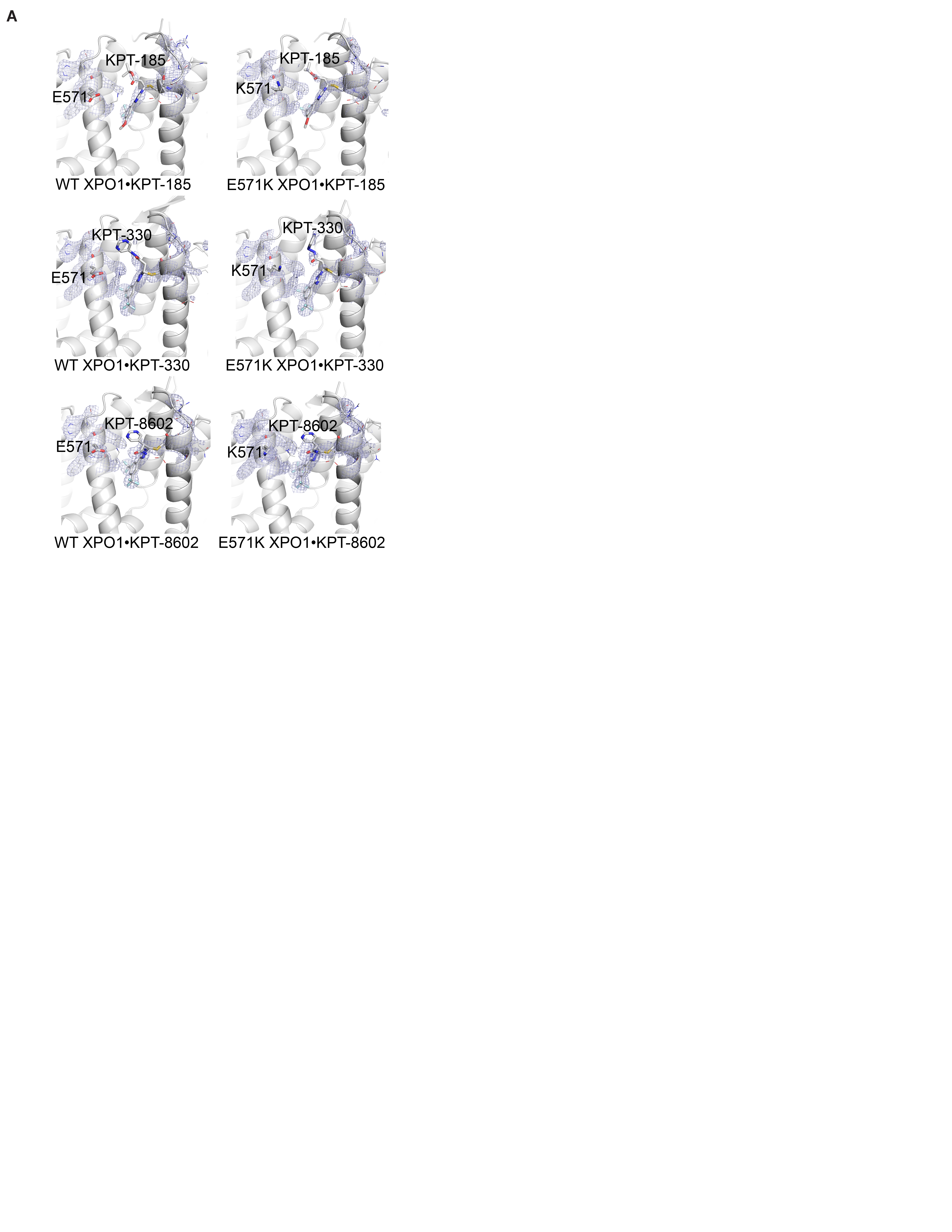

Supplement: Supplementary file 3 — Additional file 3: Figure S3. Electron density of SINE-XPO1 structures. a Crystal structures of wildtype- (WT) and E571K XPO1 bound to KPT-185, KPT-330 (selinexor) and KPT-8602 (eltanexor) are shown with electron density (blue mesh) from composite omit maps contoured to 1.0 sigma. [file 13045_2021_1032_MOESM3_ESM.tif]
