# Supplementary material for: Recurrent XPO1 mutations alter pathogenesis of chronic lymphocytic leukemia
Source: J Hematol Oncol. 2021 Jan 15;14:17. doi: 10.1186/s13045-021-01032-2 (PMC7809770; doi:10.1186/s13045-021-01032-2)
Supplement: Supplementary file 4 — Additional file 4: Table S1. IGH gene usage in Eμ-XPO1 and Eμ-XPO1xTCL1 mice. [file 13045_2021_1032_MOESM4_ESM.docx]

**Supplementary Table S1: IGH gene usage in Eμ-XPO1 and Eμ-XPO1xTCL1 mice**

| **Eµ-XPO1^WT^** | |  |  |  |  |  |  |  | **Eµ-XPO1^E571K^** | |  |  |  |  |  |  |
| --- | --- | --- | --- | --- | --- | --- | --- | --- | --- | --- | --- | --- | --- | --- | --- | --- |
| Mouse 1 |  |  | Mouse 2 |  |  | Mouse 3 |  |  | Mouse 1 |  |  | Mouse 2 |  |  | Mouse 3 |  |
| IGHV6-6 | 1.6% |  | IGHV1-50 | 1.8% |  | IGHV1-71 | 1.8% |  | IGHV8-12 | 0.3% |  | IGHV1-15 | 0.7% |  | IGHV1-55 | 1.0% |
| IGHV1-52 | 1.7% |  | IGHV6-6 | 1.9% |  | IGHV5-6 | 2.6% |  | IGHV3-6 | 0.3% |  | IGHV1-72 | 0.7% |  | IGHV4-1 | 1.1% |
| IGHV6-3 | 1.9% |  | IGHV1-80 | 2.7% |  | IGHV1-62-2 | 2.9% |  | IGHV6-3 | 0.4% |  | IGHV5-17 | 0.8% |  | IGHV1-80 | 1.3% |
| IGHV1-14 | 2.0% |  | IGHV1-72 | 3.2% |  | IGHV6-3 | 3.6% |  | IGHV9-3 | 0.4% |  | IGHV9-1 | 0.8% |  | IGHV5-9-1 | 1.6% |
| IGHV5-6 | 2.3% |  | IGHV5-6 | 4.3% |  | IGHV11-2 | 4.6% |  | IGHV1-80 | 0.5% |  | IGHV6-3 | 0.9% |  | IGHV1-7 | 1.8% |
| IGHV5-17 | 2.3% |  | IGHV3-6 | 4.3% |  | IGHV5-4 | 4.9% |  | IGHV6-6 | 0.6% |  | IGHV5-6 | 1.3% |  | IGHV6-3 | 2.0% |
| IGHV14-2 | 2.6% |  | IGHV1-55 | 7.6% |  | IGHV9-3 | 6.0% |  | IGHV1-58 | 0.7% |  | IGHV11-2 | 1.4% |  | IGHV11-2 | 2.7% |
| IGHV1-54 | 3.1% |  | IGHV6-3 | 8.6% |  | IGHV1-47 | 6.1% |  | IGHV1-55 | 1.3% |  | IGHV9-3 | 1.8% |  | IGHV1-53 | 4.1% |
| IGHV9-3 | 4.6% |  | IGHV2-9-1 | 10.6% |  | IGHV1-53 | 7.8% |  | Others | 3.8% |  | IGHV5-9 | 10.0% |  | IGHV2-5 | 4.2% |
| IGHV1-53 | 6.6% |  | IGHV1-53 | 11.6% |  | IGHV14-4 | 9.7% |  | IGHV1-53 | 15.6% |  | Others | 11.3% |  | IGHV9-4 | 9.8% |
| IGHV1-26 | 27.8% |  | IGHV9-3 | 14.7% |  | IGHV1-52 | 14.5% |  | IGHV1-15 | 27.6% |  | IGHV13-2 | 34.6% |  | Others | 11.6% |
| Others | 43.5% |  | Others | 28.6% |  | Others | 35.4% |  | IGHV5-9 | 48.7% |  | IGHV9-4 | 35.9% |  | IGHV1-26 | 58.7% |
|  |  |  |  |  |  |  |  |  |  |  |  |  |  |  |  |  |
| **Eµ-XPO1^E571G^** | |  |  |  |  |  |  |  | **C57BL/6 non-transgenic** | | |  |  |  |  |  |
| Mouse 1 |  |  | Mouse 2 |  |  | Mouse 3 |  |  | Mouse 1 |  |  | Mouse 2 |  |  | Mouse 3 |  |
| IGHV6-6 | 1.2% |  | IGHV1-19 | 1.3% |  | IGHV1-62-2 | 2.2% |  | IGHV4-1 | 2.0% |  | IGHV5-6 | 2.6% |  | IGHV8-12 | 2.4% |
| IGHV5-4 | 1.3% |  | IGHV1-75 | 1.4% |  | IGHV1-72 | 2.4% |  | IGHV1-15 | 2.0% |  | IGHV1-80 | 2.7% |  | IGHV5-4 | 2.4% |
| IGHV1-9 | 1.4% |  | IGHV1-62-2 | 1.7% |  | IGHV1-75 | 3.7% |  | IGHV5-16 | 2.2% |  | IGHV1-75 | 2.8% |  | IGHV6-6 | 2.7% |
| IGHV5-9-1 | 1.9% |  | IGHV1-59 | 1.7% |  | IGHV6-3 | 4.0% |  | IGHV1-76 | 2.6% |  | IGHV5-9-1 | 3.3% |  | IGHV1-84 | 3.0% |
| IGHV3-6 | 2.0% |  | IGHV1-80 | 1.8% |  | IGHV11-2 | 4.3% |  | IGHV3-6 | 3.0% |  | IGHV1-71 | 3.8% |  | IGHV1-80 | 4.0% |
| IGHV1-39 | 2.2% |  | IGHV1-15 | 2.3% |  | IGHV5-9-1 | 4.8% |  | IGHV1-75 | 3.0% |  | IGHV1-62-2 | 5.2% |  | IGHV1-62-2 | 4.0% |
| IGHV1-53 | 2.7% |  | IGHV9-3 | 3.3% |  | IGHV1-15 | 6.1% |  | IGHV2-3 | 3.0% |  | IGHV1-77 | 5.9% |  | IGHV1-18 | 4.2% |
| IGHV4-1 | 3.0% |  | IGHV1-9 | 3.5% |  | IGHV1-19 | 9.4% |  | IGHV6-3 | 4.0% |  | IGHV1-78 | 6.1% |  | IGHV6-3 | 6.2% |
| IGHV2-2 | 4.0% |  | IGHV3-6 | 3.5% |  | IGHV5-9 | 10.4% |  | IGHV1-53 | 6.0% |  | IGHV1-55 | 6.4% |  | IGHV1-9 | 6.9% |
| IGHV2-3 | 12.8% |  | IGHV1-55 | 13.3% |  | IGHV5-6 | 11.1% |  | IGHV5-4 | 17.3% |  | IGHV1-53 | 6.7% |  | IGHV1-53 | 7.5% |
| Others | 13.3% |  | Others | 18.2% |  | IGHV9-3 | 11.4% |  | IGHV9-3 | 18.3% |  | IGHV9-3 | 10.7% |  | IGHV9-3 | 12.1% |
| IGHV1-52 | 54.1% |  | IGHV1-18 | 48.0% |  | Others | 30.3% |  | Others | 36.7% |  | Others | 43.7% |  | Others | 44.7% |
|  |  |  |  |  |  |  |  |  |  |  |  |  |  |  |  |  |
|  |  |  |  |  |  |  |  |  |  |  |  |  |  |  |  |  |
| **Eµ-XPO1^WT^xTCL1** | |  |  |  |  |  |  |  | **Eµ-XPO1^E571K^xTCL1** | |  |  |  |  |  |  |
| Mouse 1 |  |  | Mouse 2 |  |  | Mouse 3 |  |  | Mouse 1 |  |  | Mouse 2 |  |  | Mouse 3 |  |
| IGHV5-9 | 0.0% |  | IGHV1-66 | 0.0% |  | IGHV5-4 | 0.0% |  | IGHV1-62-2 | 0.1% |  | IGHV1-14 | 0.0% |  | IGHV8-12 | 0.0% |
| IGHV11-2 | 0.1% |  | IGHV5-12 | 0.0% |  | IGHV14-1 | 0.0% |  | IGHV6-6 | 0.1% |  | IGHV2-2 | 0.0% |  | IGHV1-53 | 0.0% |
| IGHV12-3 | 0.2% |  | IGHV1-15 | 0.1% |  | IGHV1-74 | 0.0% |  | IGHV10-3 | 0.1% |  | IGHV1-78 | 0.0% |  | IGHV1-14 | 0.0% |
| IGHV2-6 | 2.6% |  | IGHV9-2 | 0.1% |  | IGHV11-2 | 0.0% |  | IGHV6-3 | 0.1% |  | IGHV9-3 | 0.0% |  | IGHV1-21 | 0.0% |
| IGHV1-74 | 97.1% |  | IGHV1-81 | 0.1% |  | IGHV5-17 | 0.0% |  | IGHV1-15 | 0.1% |  | IGHV1-66 | 0.0% |  | IGHV11-2 | 0.0% |
|  |  |  | IGHV1-5 | 0.1% |  | IGHV1-54 | 0.0% |  | IGHV5-9-1 | 0.3% |  | IGHV1-53 | 0.0% |  | IGHV12-3 | 2.5% |
|  |  |  | IGHV5-17 | 0.1% |  | IGHV7-1 | 0.2% |  | Others | 0.3% |  | IGHV5-4 | 0.0% |  | IGHV1-81 | 97.4% |
|  |  |  | IGHV1-62 | 0.1% |  | IGHV1-72 | 0.7% |  | IGHV1-9 | 0.4% |  | IGHV14-4 | 0.1% |  |  |  |
|  |  |  | IGHV1-14 | 0.1% |  | IGHV12-3 | 99.0% |  | IGHV11-2 | 0.5% |  | IGHV1-62-2 | 0.1% |  |  |  |
|  |  |  | IGHV13-2 | 0.1% |  |  |  |  | IGHV5-6 | 2.3% |  | Others | 0.1% |  |  |  |
|  |  |  | Others | 0.8% |  |  |  |  | IGHV12-3 | 5.1% |  | IGHV11-2 | 0.3% |  |  |  |
|  |  |  | IGHV2-9 | 98.6% |  |  |  |  | IGHV1-53 | 90.6% |  | IGHV12-3 | 99.4% |  |  |  |
|  |  |  |  |  |  |  |  |  |  |  |  |  |  |  |  |  |
| **Eµ-XPO1^E571G^xTCL1** | |  |  |  |  |  |  |  | **Eµ-TCL1** |  |  |  |  |  |  |  |
| Mouse 1 |  |  | Mouse 2 |  |  | Mouse 3 |  |  | Mouse 1 |  |  | Mouse 2 |  |  | Mouse 3 |  |
| IGHV5-4 | 0.5% |  | IGHV7-3 | 0.3% |  | IGHV1-81 | 0.0% |  | IGHV1-14 | 0.1% |  | IGHV1-82 | 0.0% |  | IGHV1-20 | 0.0% |
| IGHV5-17 | 0.5% |  | IGHV2-9-1 | 0.3% |  | IGHV2-3 | 0.0% |  | IGHV12-3 | 0.1% |  | IGHV5-12 | 0.0% |  | IGHV9-2 | 0.0% |
| IGHV4-1 | 0.6% |  | IGHV1-75 | 0.3% |  | IGHV1-62-2 | 0.0% |  | IGHV14-2 | 0.2% |  | IGHV1-74 | 0.0% |  | IGHV1-2 | 0.0% |
| IGHV1-80 | 0.7% |  | IGHV5-16 | 0.3% |  | IGHV9-3 | 0.0% |  | IGHV6-3 | 0.2% |  | IGHV1-52 | 0.0% |  | IGHV1-9 | 0.0% |
| IGHV3-6 | 1.0% |  | IGHV6-6 | 0.3% |  | IGHV9-2 | 0.0% |  | IGHV1-72 | 0.2% |  | IGHV14-1 | 0.0% |  | IGHV5-17 | 0.0% |
| IGHV1-53 | 1.1% |  | IGHV6-3 | 0.4% |  | IGHV1-63 | 0.0% |  | IGHV5-17 | 0.3% |  | IGHV5-9-1 | 0.0% |  | IGHV1-19 | 0.0% |
| IGHV8-12 | 1.6% |  | IGHV8-5 | 0.4% |  | IGHV2-9-1 | 0.0% |  | IGHV5-9-1 | 0.5% |  | IGHV1-50 | 0.0% |  | IGHV1-55 | 0.2% |
| IGHV1-64 | 2.3% |  | IGHV1-15 | 0.7% |  | Others | 0.0% |  | IGHV1-15 | 1.4% |  | IGHV1-14 | 0.0% |  | IGHV1-18 | 99.7% |
| IGHV11-2 | 5.1% |  | IGHV9-3 | 0.8% |  | IGHV5-4 | 0.1% |  | Others | 1.7% |  | Others | 0.0% |  |  |  |
| IGHV12-3 | 5.4% |  | IGHV10-1 | 0.9% |  | IGHV1-82 | 0.1% |  | IGHV11-2 | 3.1% |  | IGHV1-81 | 0.0% |  |  |  |
| Others | 7.5% |  | Others | 5.2% |  | IGHV12-3 | 37.5% |  | IGHV1-71 | 25.4% |  | IGHV6-3 | 0.0% |  |  |  |
| IGHV1-54 | 73.8% |  | IGHV1-84 | 90.0% |  | IGHV11-2 | 62.0% |  | IGHV1-63 | 67.0% |  | IGHV12-3 | 99.9% |  |  |  |
